# Genetic marker: a genome mapping tool to decode genetic diversity of livestock animals

**DOI:** 10.3389/fgene.2024.1463474

**Published:** 2024-10-17

**Authors:** Darshan C. Panchariya, Priyanka Dutta, Ananya, Adyasha Mishra, Aakash Chawade, Nilesh Nayee, Sarwar Azam, Ravi Kumar Gandham, Subeer Majumdar, Sandeep Kumar Kushwaha

**Affiliations:** ^1^ National Institute of Animal Biotechnology, Hyderabad, India; ^2^ Department of Biochemistry and Molecular Biophysics, Washington University in St. Louis, St. Louis, MO, United States; ^3^ Center for Life Sciences, Mahindra University, Hyderabad, India; ^4^ Department of Plant Breeding, Swedish University of Agricultural Sciences, Alnarp, Sweden; ^5^ National Dairy Development Board, Anand, Gujarat, India; ^6^ Indian Institute of Technology Hyderabad, Hyderabad, India; ^7^ ICAR-National Bureau of Animal Genetic Resources, Karnal, India; ^8^ Gujarat Biotechnology University, Gandhinagar, Gujarat, India

**Keywords:** genome, genotyping, genetic markers, genetic diversity, breeding, marker-assisted breeding

## Abstract

Genotyping is the process of determining the genetic makeup of an organism by examining its DNA sequences using various genetic markers. It has been widely used in various fields, such as agriculture, biomedical and conservation research, to study genetic diversity, inheritance, the genetic basis of disease-associated traits, evolution, adaptation, etc., Genotyping markers have evolved immensely and are broadly classified as random markers (RFLP, RAPD, AFLP, etc.) and functional markers (SCoT, CDDP, SRAP, etc.). However, functional markers are very limited in genotype studies, especially in animal science, despite their advantages in overcoming the limitations of random markers, which are directly linked with phenotypic traits, high specificity, and similar logistic requirements. The current review surveyed the available random and functional markers for genotyping applications, focusing on livestock including plant and microbe domains. This review article summarises the application, advantages, and limitations of developed markers and methods for genotyping applications. This review aims to make the reader aware of all available markers, their design principles, and methods, and we discuss the marker inheritance patterns of RLFP and AFLP. The review further outlines the marker selection for particular applications and endorses the application of functional markers in genotyping research.

## Highlights

• The genotyping techniques enable us to explore the wealth of information encoded within the genome.
• Various DNA markers have been developed. However, only some DNA markers are widely used in human, animal, and plant research due to their theoretical and technical advantages and capacity for sample processing and analysis.
• This review provides an overview of all known markers and methods for genotyping studies. It summarises their potential for application, advantages, limitations, and inheritance patterns to choose markers for genotyping applications.
• Various functional markers have been developed and widely used in plant science. However, it is underutilised in animal science despite several advantages over random markers.

## 1 Introduction

A gene consists of nucleotide sequences encompassing the information to encode functional molecules like RNA and proteins to shape phenotypic traits of organisms (Gerstein et al., 2007). These genes and gene regulatory regions can have minor differences in nucleotide sequences in a population, known as genetic polymorphism, representing the variation in individual genome sequences within and between species (Saeb and Al-Naqeb, 2016; Jiang, 2013). These genomic variations can alter the genetic potential of an individual in the population. Therefore, high-quality, informative markers are essential to differentiate the genomic merits of existing genetic resources and are required to correlate the phenotypic performance of individual organisms in a population (Saeb and Al-Naqeb, 2016). Biological molecules, such as DNA, RNA, protein, metabolite, etc., may be used as markers if these markers can measure specific phenotypic differences between samples accurately and reproducibly (Jiang, 2013). The current review delves into the intricacies of molecular markers, their principles, pros and cons, and selective applications, with a major focus on livestock application. Further, for the first time, we outline the probable mechanism behind the differential marker inheritance in RFLP and AFLP, which are technically similar markers.

## 2 Biological markers for genetic diversity studies

**FIGURE 1:**
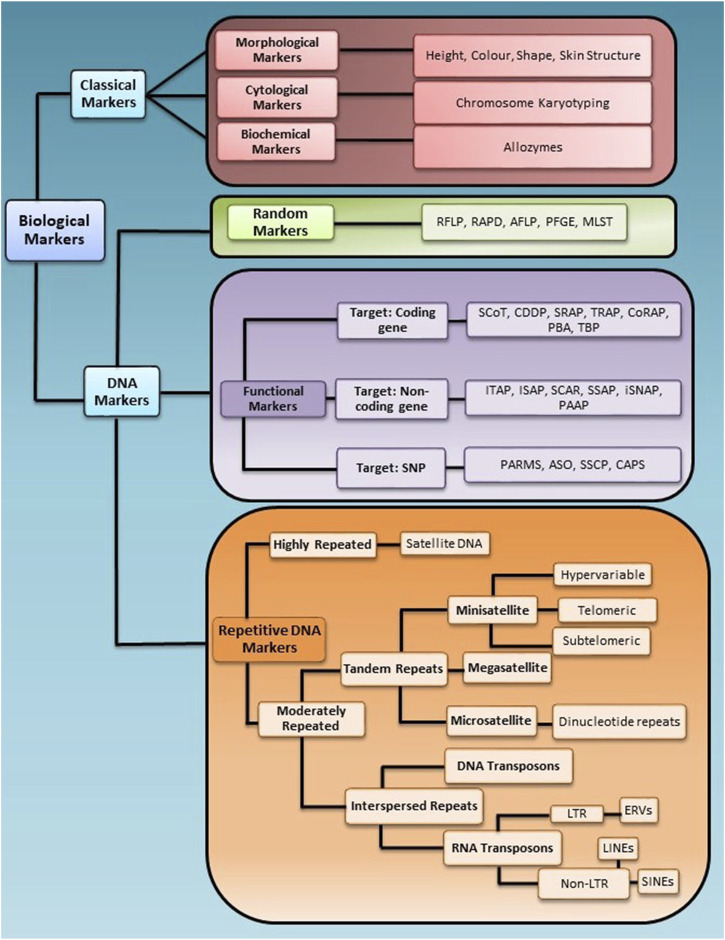
Schematic representation of marker classification for the application in genotyping studies. Biological markers are broadly divided into two categories, Classical and DNA markers, further divided into sub-categories based on their nature of targeting the genome.

Selecting the appropriate marker, marker type, and number is essential to get a high genotypic resolution between individuals in a population. Based on the application, biological markers are grouped into two broad categories, i.e., classical markers and molecular markers. A brief schematic representation of marker classification is given in Figure 1.

### 2.1 Classical markers

Classical markers are the most primitive markers that use identifiers such as morphology, the cytological features or the biochemical features distinct to an organism to genotype; some classical markers include morphological markers, cytological markers and biochemical or protein markers. However, these markers are highly variable to environmental changes and do not accurately map the genetic basis of a trait. Therefore, genetically stable and inheritable markers based on DNA were developed.

### 2.2 DNA markers

DNA markers are sets of nucleotide bases of DNA sequence that exhibit polymorphism to differentiate individuals of a population (Jiang, 2013). DNA markers have several advantages over other markers, such as direct links to genetic changes and no influence on tissue type, environment, or growth condition. These are desirable features of ideal makers for marker-assisted selection strategies (Hasan et al., 2021; Yang et al., 2013). The concept of using a gene as a unit of selection for population studies was first reported in Clegg et al. (1972). Later in the 1980s, molecular markers became popular and applied in various crop plants for QTLs mapping to assess genetic gain in plant breeding research (Clegg et al., 1972; Paterson et al., 1990; Soller and Beckmann, 1983; Young and Tanksley, 1989).

Due to their high resolution and stability potential, various DNA-based markers were developed and used in the 1980s. DNA markers have several advantages over conventional markers for genotyping studies, such as no detrimental effect on the phenotype, easy detection methods (PCR-based or hybridization-based markers), high marker polymorphism, whole genome-based maker analysis, high reproducibility, cost-effective and high automation possibilities and easy marker inheritance analysis (dominant or co-dominant, locus-specific or non-locus-specific).

## 3 Random DNA markers for genetic diversity studies

The selection of genotyping markers and methods depends on the number of samples and markers, and an adequate number is required to get resolution at the genome level. This section summarises the principle, procedure, and analysis of various random DNA markers for genotyping and their application in biological sciences. A schematic representation of the detailed protocol of each marker protocol is provided as (Supplementary Figures S1–S3). DNA random makers are handy when genome sequence information is unavailable, and few samples must be compared for genetic diversity.

### 3.1 Restriction fragment length polymorphism (RFLP)

Background: Botstein et al. (1980) constructed the first human genetic linkage map using RFLP in 1980*.* RFLP works on the principle restriction enzyme (RE) digestion that produces DNA fragments of different sizes due to the polymorphism at the restriction site, which occurs due to a loss or gain and alteration of genome sequences at the restriction site (Botstein et al., 1980). In the event of loss of recognition site, a longer fragment shall be generated, whereas there would be two fragments if the gain is in restriction site number.

Pros and cons: RFLP markers are co-dominant and locus-specific. These are easy to use and have high reproducibility. PCR-RFLP is an advanced version of RFLP markers, also known as Cleaved Amplified Polymorphism Sequences (CAPS). RFLP markers have several disadvantages, such as inconsistent resolution, requiring sequence information to interpret complex allelic variants, low genotyping throughput, and the need for high-quality DNA and radioactive probes, which restrict its wide-scale application (Jiang, 2013).

Applications: Naufal and Noor, (2024) apply PCR-RFLP to categorise the breed-specific polymorphism in five beef cattle breeds of Indonesia. Targeting the Exon1 of the myostatin gene, the authors could distinguish breed-specific polymorphic signature of isolation, i.e., the SNP 111G>C was found in two cattle breeds (Madura and PO). In comparison, an SNP 276G>A was found in Madhura, Bali and PO. They found two cattle breeds to be monomorphic for this gene (Simmental and Limousin). The RFLP markers were helpful in the estimation of heterozygosity, genotype and allele frequencies, aiding in the genetic characterization of livestock population (Naufal and Noor, 2024).

### 3.2 Random amplified polymorphic DNA (RAPD)

Background: RAPD markers were developed in the 1990s for constructing genetic maps. The RAPD method is simple and has a higher throughput than the RFLP technique. Performing an RAPD assay does not require sequence information and relies on a single arbitrary primer for detection. RAPD principle lies in detecting polymorphism using these primers without specific nucleotide sequences, which can help construct genetic maps using the polymorphic markers (Williams et al., 1990). RAPD markers are dominant and non-locus-specific.

Pros and cons: RAPD is easy to perform, requires less DNA (i.e., 10 ng per reaction), quick result visualisation, no sequence information needed and has high automation possibility due to PCR-based assay. The protocol does not involve the tedious procedure of southern blotting using harmful radioactive probes. The primers are not species-specific, and the amplification product can be cloned for sequence information. However, the markers have several disadvantages, such as non-specific sequences, which may lead to error-prone result interpretation, low reproducibility, and inability to differentiate allelic differences in heterozygotes (Jiang, 2013).

Applications: Tayde et al. (2024) exploit the universality of RAPD markers using a combination of 12 oligonucleotide primers in differentiating Indian cattle breeds. The authors successfully identified 8 polymorphic markers and 4 monomorphic markers, with an observation that the polymorphic markers showed relatedness among the selected breeds (Tayde et al., 2024).

### 3.3 Amplified fragment length polymorphism (AFLP)

Background: In Vos et al. (1995) combined the PCR and RFLP techniques to improve the genotyping resolution, known as AFLP. It comprises three steps: RE digestion of DNA, adapter ligation for PCR amplification, and electrophoresis. The primers are designed for the known adapter sequences to amplify restriction fragments. These restriction fragments can be selectively amplified and visualised on denaturing acrylamide gel (Vos et al., 1995). AFLP markers are dominant and locus-specific.

Pros and cons: AFLP does not require prior knowledge of sequence information to design specific probes; AFLP allows bias-free species diversity identification like SNP-based genotyping. A group of REs can produce thousands of unique AFLP fragments for whole genome QTL mapping studies. AFLP markers further overcome the complexity associated with RFLP and the reliability and reproducibility of RAPD methods. However, AFLP markers are dominant bi-allelic markers. Therefore, they fail to differentiate the dominant homozygous from the dominant heterozygous individuals (Sharma et al., 2006; Utsunomiya et al., 2014; Yang et al., 2013).

Applications: In animal science, AFLP markers have been used for various diversity and quantitative trait studies, such as marker-assisted selection to identify QTLs linked to mastitis disease resistance. This study was conducted on 100 animals each for the clinical mastitis disease-resistant and susceptible group and identified 27 AFLP markers with a false discovery rate of <5%. One of these markers (CGIL4) has a higher allele frequency in the clinically resistant group, which shows the occurrence of single nucleotide polymorphism (A↔G) (Sharma et al., 2006). AFLP markers have also complemented other high-throughput genotyping methods, such as understanding the genomic architecture of Zebu and Taurine cattle. SNP markers are well-optimised for taurine cattle, but there is limited information on Zebu cattle. Therefore, AFLP markers significantly contributed to understand Zebu cattle’s genetic structure and diversity (Utsunomiya et al., 2014).

Livestock production can be severely affected by infectious diseases, and therefore, the following section discusses the methods available for microbial identification and genotyping, (Supplementary Figures S4–S8) describe the methods in detail.

### 3.4 Pulse field gel electrophoresis (PFGE)

Background: The PFGE method was developed in 1984 by Schwartz and Cantor. This method is based on RE digestion and alternating pulsed electric fields passed through a perpendicularly oriented gel. It separates high molecular weight DNA with a higher resolution than traditional electrophoresis. It fractionates the DNA into several bands, producing a molecular karyotype for microorganisms.

Pros and cons: PFGE is a variant of agarose gel electrophoresis that permits analysis of a large bacterial DNA fragment of a higher magnitude than conventional restriction enzyme analysis. PFGE provides a reproducible restriction profile and good coverage of the entire chromosome of bacteria in a single gel as a distinct and well-resolved DNA fragment (Schwartz and Cantor, 1984). One of the disadvantages associated with PFGE is the time involved in analysing the results, which takes three to 4 days (Magalhães et al., 2014).

Applications: Shoaib et al. (2023) describe the application of PFGE in constructing a phylogenetic map of non-aureus *Staphylococcus* species, *Staphylococcus haemolyticus* from dairy cattle milk. They identified 36 *S. haemolyticus* strains by PFGE patterns generated after digestion with the *Sma I* restriction enzyme. These 39 strains were clustered into eight phylogenetic groups (Shoaib et al., 2023).

### 3.5 Multi-locus sequence typing (MLST)

Background: In 1998, the MLST method was developed for molecular epidemiology of *Neisseria meningitides*, which is highly advanced today. It is a modification of the multi-locus enzyme electrophoresis (MLEE) technique that identified the neutral mutations that accumulate over time in the housekeeping enzymes. MLST directly identifies the variation in the nucleotide sequence of the housekeeping genes’ alleles instead of their enzymes’ electrophoretic properties. The relatedness between isolated types is shown by a dendrogram constructed using the distance matrix of their allelic profiles (Enright et al., 1999; Maiden et al., 1998).

Pros and cons: This method enables the identification of more variations than the original MLEE method. The allelic profiles of the MLST analysis can be directly compared in a central database via the Internet. In the absence of sequence information, housekeeping genes can be used for PCR amplification for allelic profiling (Multi-Locus Sequence Typing, 2023). Despite its potential in discriminating individual bacterial isolates, this method relies on accumulating nucleotide changes in the housekeeping genes, which is a relatively slow process. Additionally, it generates a large amount of data, requiring skilled personnel for data analysis (Enright et al., 1999).

Applications: MLST can be beneficial in identifying specific isolates of microbes, such as *Klebsiella pneumoniae*, that threaten humans, cattle, and other animals. Cheng et al. (2018) collected nasal swabs of 213 sick cows suffering from respiratory manifestations and confirmed *K. pneumoniae* as a pathogen using 16s rRNA primers. *K. pneumonia* isolates were tested for antibiotic sensitivity and virulence-associated gene profile using MLST. In this study, thirty-three isolates of *K. pneuomniae* were identified, of which twelve were hyper-virulent. Antimicrobial resistance for β-lactamases was observed in 93.4% of the studied strains (Cheng et al., 2018). Sivakumar et al. (2023) investigated 41 mastitis-associated strains of *Staphylococcus aureus* isolated from India. This study identified 15 diverse sequence types and five clonal complexes (CC) using genome sequencing and MLST. The study also reported the *S. aureus strain MUF256* as the common ancestor of all the genomic isolate sequences of India, and all the Indian *S. aureus* isolates belonging to the CC97 are mastitis-associated. The study also reported 17 antimicrobial resistance (AMR) genes and 108 virulence-associated genes among isolates (Sivakumar et al., 2023).

### 3.6 Ribotyping

Background: In 1986, ribotyping was developed for strain typing using rRNA banding patterns. The protocol of ribotyping is similar to RFLP, except for hybridisation with a universal conserved rRNA probe. The banding pattern produced through hybridised rRNA probes is known as ribotype, which reveals rRNA loci-associated polymorphism and positions. The ribosomal polymorphism pattern is strain-specific genotyping (Grimont and Grimont, 1986).

Pros and cons: It is based on universal rRNA probes, making its application simple and highly reproducible. It is a laborious, time-consuming process with lower discriminatory power than pulsed-field gel electrophoresis (Ramadan, 2022; Snipes et al., 1989).

Applications: Zhang et al. (2020) mention the application of PCR-Ribotyping in identifying *Clostridium difficille* and found 55 *C. difficille* isolates from 953 animal stool samples. Among these, 51 strains were from newborn calves aged less than 7 days. Six ribotypes (ICDC028, n = 2; ICDC035, n = 35; ICDC039, n = 1; ICDC050, n = 8; ICDC052, n = 6 and ICDC094, n = 3) were identified, among which RT126 (ICDC028) was the predominant type and had high antibiotic resistance (Zhang et al., 2020).

### 3.7 Spoligotyping

Background: Spacer oligonucleotide typing (Spoligotyping) is a method developed by Kamerbeek et al. in the 1990s. It detects the presence or absence of spacers of known sequence in an isolate in two steps. First, PCR is used to amplify the regions between the direct repeats (DR), using a biotinylated reverse primer during PCR; the reverse strand is labelled; second, the direct repeat sequence variability by hybridisation (Aranaz et al., 1996). Spoligotyping markers are dominant and locus-specific.

Pros and cons: Spoligotyping is a rapid method of identifying *Mycobacterium* and hence used in genotyping the *Mycobacterium* complex. However, it has limited discriminatory power (only lineage or sub-lineage levels) and cannot discriminate at the strain level. Variability in the spoligotyping membranes can cause errors in the spoligotype patterns interpretation.

Applications: The spoligotyping technique was developed on *Mycobacterium*’s direct repeat (DR) elements. Therefore, it has excellent application in genotyping the *Mycobacterium tuberculosis* complex. Sahin et al. describe its application in genotyping *Mycobacterium bovis*. Thirty-eight isolates of *M. bovis* were spoligotyped and discriminated into four different groups (Merker et al., 2017).

### 3.8 BOX/ERIC/REP-PCR

This method is based on analysing conserved repetitive DNA elements, such as Repetitive Extragenic Palindromic (REP) regions, Enterobacterial Repetitive Intergenic Consensus (ERIC) sequence, found in *E. coli* and *S. typhimurium* (Gilson et al., 1984)*,* used as molecular tools to genotype bacteria (Versalovic et al., 1991). REP elements are 38 bp sequences containing a 5 bps variable loop between each side of a conserved palindromic stem. ERIC sequences are highly conserved central inverted long (126-bps) sequence repeats located in extragenic regions of the bacterial genome. BOX-PCR analysis is based on the BOX dispersed-repeat motif in the intergenic regions of the genome. These are mosaic repetitive elements composed of combinations of three (boxA, boxB, and boxC) subunit sequences identified in *Streptococcus pneumoniae* bacterial species. Primers designed based on BOX/ERIC/REP sequence information help to identify strain-specific bacteria (Olive and Bean, 1999).

Pros and cons: It is an easy, low-cost, efficient technique that is scalable for many samples. BOX elements are interspersed throughout the bacterial genome. REP-PCR has higher discriminatory power than ribotyping and multi-locus enzyme electrophoresis but lower discriminatory power than PFGE. However, this technique requires a standardisation run to compare the bands in different gels and the issues of poor band resolution. Fluorescent BOX-PCR (F-BOX PCR) has been developed to overcome these issues (Brusetti et al., 2008; Tabit, 2016).

**FIGURE 2:**
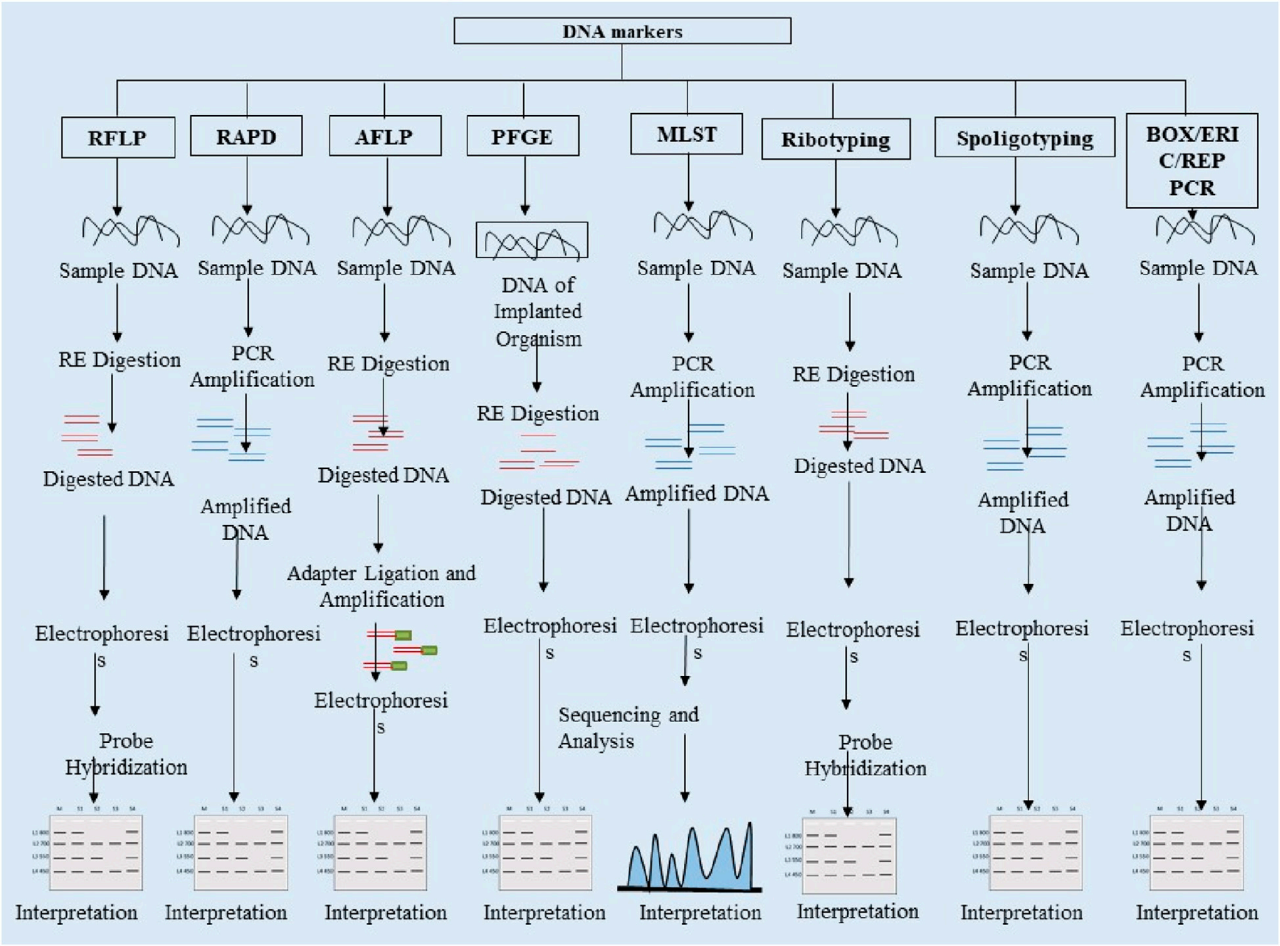
Schematic representation of overview of molecular techniques based on DNA markers.

Applications: ERIC-PCR, one of the reliable techniques, has been used in various studies on bacterial genotyping. Sielski et al. used it to characterise the antibiotic resistance of *Salmonella heidelber*g in Brazil, and 130*S. heidelergs* isolates were isolated. ERIC-PCR-based dendrogram clustered the isolates into 27 clusters (Sielski Galvão Soares et al., 2023). Figure 2 compiles the schematic representation of the workflow of marker analysis for the Random DNA markers and Table 1 compares commonly used markers and their features.

**TABLE 1:** Comparative table of the most commonly used molecular markers/methods and their attributed features.

Marker/Method	DP	Typ (%)	Reproducibility	SGI	Time	Cost	AY	MI
RFLP	Good	100	High	Yes	A week	Expensive	Good	Co-dominant
AFLP	Moderate	85–90	High	Yes	2-3 days	Average	Complex method	Dominant
RAPD	Average	80	Low	Yes	<1 day	Low	Good	Dominant
PFGE	Good	100	High	Yes	3-4 days	Average	Limited	NA
MLST	Moderate	80–90	High	Yes	1-2 days	Expensive	Limited	NA
RIBOTYPING	Poor	100	Moderate	Yes	3-4 days	Average	Complex method	NA
AUTOMATIC RIBOTYPING	Good	NA	Moderate	Yes	8 h	Expensive	Limited	NA
SPOLIGOTYPING	Good	94%	Moderate	Yes	1 day	Low	Good	NA
REP/ERIC/BOX PCR	Good	NA	Moderate	Yes	1 day	Low	Good	NA

DP, *discriminatory* power; Typ, Typeability (%); SGI, sensitivity to genetic instability; AY, availability; MI, marker inheritance; NA, not applicable.

## 4 What do the inheritance patterns of markers dominant and co-dominant mean?

**FIGURE 3:**
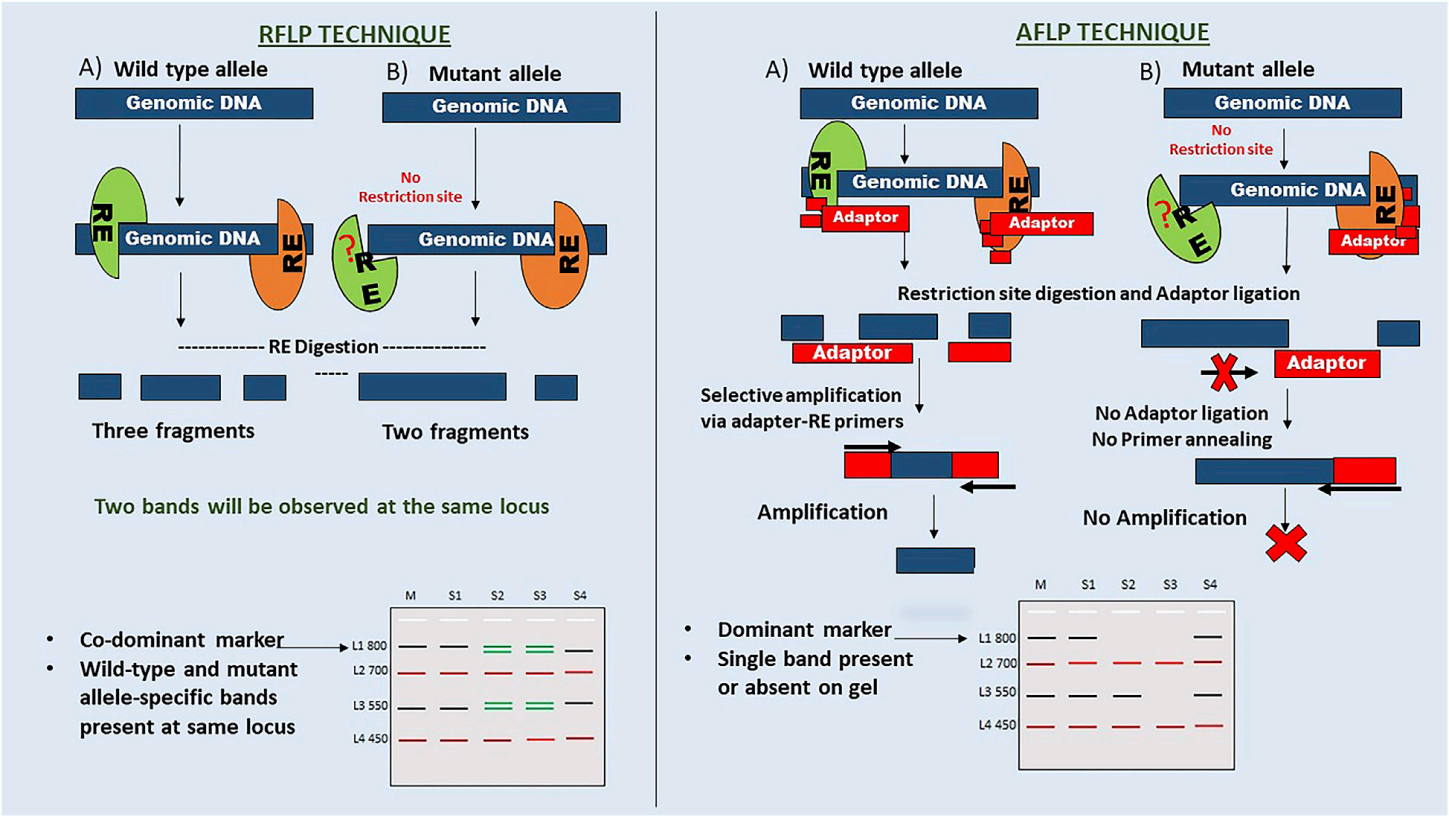
Comparison of maker inheritance. The figure highlights the fundamental procedure of RFLP and AFLP techniques. Here, the inheritance pattern is explained through a hypothetical gel image with four samples labelled S1-S4 and four loci with the wild type or mutant alleles (L: ladder, M: Marker). RFLP: In this case, there are two mutant alleles at L1 and L3, showing loss of sequence and gain of sequence mutations (i.e. size), respectively. S2 and S3 samples show co-dominant bands at these two loci, while S4 shows only mutant specific. L2 and L4 are monomorphic loci. AFLP: In the hypothetical gel, the presence or absence of a band at L1 and L3 for these samples indicates a dominant marker inheritance. It does not explain the heterozygosity of the loci for the allele. This mechanism suggests that adaptor ligation and primer annealing are responsible for the dominant marker inheritance of the AFLP technique.

DNA of an offspring is inherited from parents, which can be recognised by similarities and differences in their DNA sequences. These differences in DNA sequence may be a part of a gene or may not have a known function, leading to DNA polymorphism (Amiteye, 2021). As per Mendelian genetics, genes exist in biallelic form, wild-type and mutant. Allelic forms of genes (dominant or co-dominant markers) can be used to understand marker inheritance. The pattern analysis of marker inheritance can be used to understand the organism’s genetics. Here, marker inheritance is explained using a hypothetical analysis of RFLP (co-dominant markers) and AFLP (dominant markers) markers on a hypothetical DNA sequence, as shown in Figure 3. As per Mendelian genetics, F2 generation produces offspring in the ratio of 1:2:1 (homozygous dominant: heterozygous dominant: homozygous recessive) alleles as a result of a homozygous wild-type and mutant cross, followed by F1 self-crossing. While RFLP markers can differentiate homozygous and heterozygous alleles, AFLP markers failed to do so. Thus, markers that can differentiate pure homozygous dominants from heterozygous dominants are called co-dominant markers (RFLP). Markers that fail to differentiate homozygous and heterozygous dominant are known as dominant markers (AFLP). The mechanism of co-dominant inheritance in RFLP markers can be explained by the method used in identification. They do not rely on primer-based amplification of restriction fragments for downstream processing. However, AFLP markers need to be ligated with restriction site-specific adaptors, which do not ligate in the absence of a restriction site; thus, there is no amplification and detection of the mutant allele. These markers report the presence or absence of an allele but do not differentiate homozygous dominants from heterozygous dominants.

## 5 Functional markers

DNA markers have significantly contributed to identifying the regions of interest for genotyping studies. However, polymorphic regions identified by DNA markers may not always be linked to a phenotypic trait and facilitate numerous non-specific gene introgression; desired genes may be located far away from identified regions. The introgression of non-specific genetic elements may compromise the trait selection process in marker-assisted breeding (MAB). Thus, functional markers (FMs) are an excellent alternative to random DNA markers for genotyping studies. Functional markers are derived from the genome’s functionally active regions and manifest as definite phenotypes in the progeny, facilitating selection with high precision (Collard and Mackill, 2008; Lau et al., 2015). FMs can help to avoid false positive selection and information loss while reducing the gap between genotype and phenotype.

Various parameters must be considered while developing functional markers for a species or population, such as the genome information and complexity, purpose, quality and quantity of available DNA, polymorphism level in closely related species, instrumentation, time and cost. Numerous gene and genome-based functional markers and methods have been developed in past decades for genotyping studies.

Advancements in sequencing technologies and bioinformatics have significantly enhanced the accessibility of functional markers (FM). The functional markers discovery is a multi-step procedure that commences with the selection of the target species and its subsequent molecular characterization. The vast amount of data generated through multi-omics needs to be analyzed to identify patterns of interest and pinpoint differentially expressed candidate genes and sequence variations using tools such as GATK and FreeBayes. Furthermore, they should be annotated using tools such as SnpEff and Variant Effect Predictor (VEP) (Cingolani et al., 2012; FreeBayes - Research Computing Documentation, n.d.; McLaren et al., 2016). The data analysis procedure for each of the methods is beyond the scope of the current review. However, various user-friendly pipelines such as SARTools, PRADA, TRAPLINE for transcriptomics; MetMiner, xMSanalyzer for metabolomics; FragPipeAnalyst, AlphaPept for proteomics data analysis may be used for data analysis (Hsiao et al., 2024; Strauss et al., 2024; Torres-García et al., 2014; Uppal et al., 2013; Varet et al., 2016; Wang et al., 2024; Wolfien et al., 2016).

Figure 4 illustrates a bioinformatics-led approach to functional marker discovery and validation, while (Supplementary Figures S9–S19) provide detailed descriptions and schematics of each functional marker.

**FIGURE 4:**
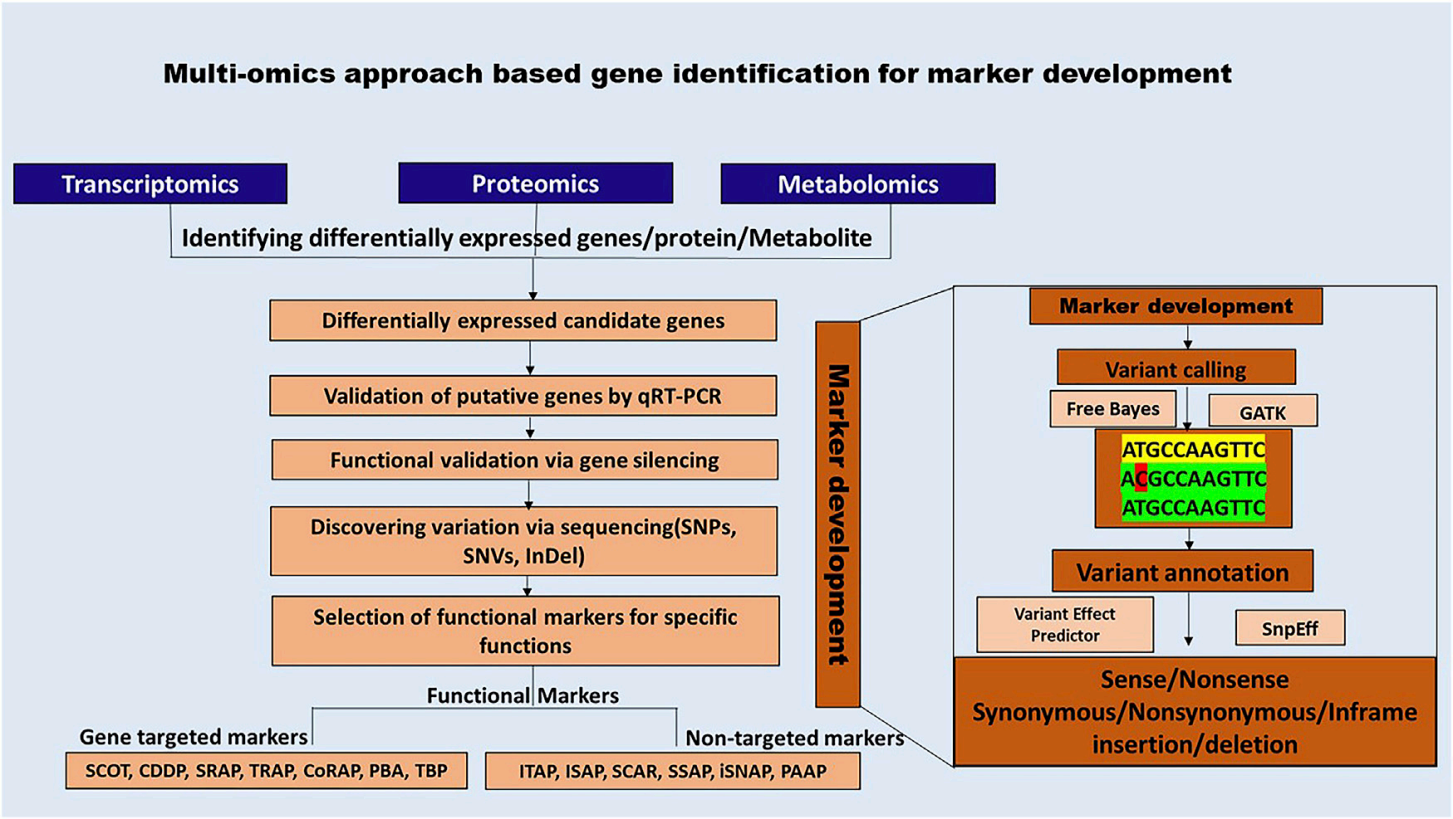
Schematic representation of multi-omics-based approach for developing functional markers. Functional markers rely on the availability of genomic information and bioinformatic analysis for variant identification, which is crucial for their development.

### 5.1 Gene targeting functional markers

#### 5.1.1 Start codon-targeted (SCoT) polymorphism

Background: Developed in 2008 by Collard and Mackill, SCoT markers target the flanking region around the start codon. Similar to RAPD, these use a single primer-based PCR amplification. SCoT primers are 18 nucleotides long and are designed to target the consensus sequence flanking start codon. The detection is based on a PCR assay and visualisation in ethidium bromide-stained agarose gel electrophoresis (Collard and Mackill, 2009b). These are dominant markers with locus specificity (Poczai et al., 2013).

Pros and cons: They have advantages like high polymorphism, simplified operation, inexpensive, reproducible, and simple primer design. However, reproducibility depends on factors such as primer length, annealing temperatures, and co-factors (Mg), which may influence reproducibility (Zeng et al., 2016).

Applications: SCoT has been applied in plant QTL mapping, bulked segregant analysis, and genetic diversity studies (Cabo et al., 2014; Gholami et al., 2021; Yeken et al., 2022). No study has been reported on farm animals in the public domain, but studies related to lower vertebrates such as sea cucumbers and fish are available. Abdelghany et al., 2023 describe the application of SCoT in sea cucumbers. SCoT was used to identify the genetic differentiation among five different species of sea cucumber in the Red Sea, Egypt, listed as *Holothuria atra, H. impatiens, H. leucospilota, Actinopyga crassa,* and *A. mauritiana*. Ten SCoT primers generated 150 amplicons with almost 52% polymorphism and 30 species-specific bands (Abdelghany et al., 2023).

#### 5.1.2 Conserved DNA-derived polymorphism (CDDP)

Background: The limitations of RAPD and other random DNA markers demanded more specificity and reproducibility. In 2009, Collard and Mackill developed CDDP markers based on single primer-mediated amplification like RAPD. CDDP uses long primers that target plants’ genes responsive to abiotic and biotic stress (Collard and Mackill, 2009a; Golian et al., 2022; Sabo et al., 2022).

Pros and cons: CDDP markers overcome the reproducibility limitations of RAPD markers because primers are gene-specific, easy to apply and can be automated. However, occasional primer-related issues, such as primer length and high annealing temperatures, may arise that fail to ensure complete reproducibility (Poczai et al., 2013). CDDP markers are dominant, but no literature-based information exists for locus specificity.

Applications: CDDP was developed using sequences conserved within the plant genome responsive to abiotic and biotic stress. CDDP has its application in genetic analysis related to polymorphism, segregant analysis, and QTL mapping. No applications are available for these animal markers; however, many studies cited them for plant-related applications. Jiang and Zang have used CDDP to analyse genetic relationships in *Rosa rugose*. The study highlights the potential of this technology as a resource in germplasm conservation, analysing a plant’s genetic relatedness (Jiang and Zang, 2018).

#### 5.1.3 Sequence-related amplified polymorphism (SRAP)

Background: The technique for SRAP markers utilises two primers of 17-18 nucleotides in length. They target the open reading frame (ORF) of the gene. The primer structure determines the specificity of this technique, consisting of a core sequence of 13-14 bases, with the first 10-11 at the 5′ end. These bases have no specific constitution; these are termed filler sequences. They are followed by the sequence CCGG (targeting GC-rich ORF) in the forward primer and AATT (Targeting the AT-rich sequences) in the reverse primer. Three additional selective bases follow the core sequence at the 3′ end. It is important to note that the filler sequences for both primers must be different.

The protocol has a distinct PCR cycle. Initially, five cycles with an annealing temperature of 35°C, followed by 35 cycles of 50°C as the annealing temperature. It is resolved on a polyacrylamide gel. SRAP markers are dominant and locus-specific (Li and Quiros, 2001; Poczai et al., 2013).

Pros and cons: Performing SRAP assay is simple, reliable, has moderate throughput potential, and is cost-effective (Aneja et al., 2012). However, only 20% of the originally developed makers were co-dominant. Overlapping bands might lead to erroneous result interpretation. The 2-step PCR reaction makes it complex. The low annealing temperature might lead to non-specific primer annealing as well.

Application: Lin et al. (2003) constructed a genetic linkage map for cotton using the SRAP markers. The study used 136 different primer pairs and found 76 of these to be polymorphic. All the markers were evenly distributed among the linkage groups without loci clustering (Lin et al., 2003).

Alasaad et al. (2008) used SRAP to identify genetic variability among Fasciola using 10 SRAP primers. These Fasciola samples clustered into four groups but were unrelated to particular host species or the samples’ geographical locations. This study highlighted the application of SRAP for genetic diversity studies of parasites significant for human-animal health (Alasaad et al., 2008).

#### 5.1.4 Target region amplification polymorphism (TRAP)

Background: In 2003, Hu and Vick developed TRAP using bioinformatics tools and EST database information to generate polymorphic markers around targeted gene sequences. Like SRAP, the method for TRAP relies on two primers of 18 nucleotides in length, except one is fixed and derived from EST, the other an arbitrary primer with an AT-rich (intron targeted) or GC-rich (exon targeted) core. It has two PCR cycles, one at 35°C for five cycles and another at 50°C. The bands are separated on a 6.5% polyacrylamide gel (Hu and Vick, 2003). TRAP is a dominant marker and is highly locus-specific.

Pros and cons: It has a simplified primer design step based on available sequence information. Samples do not require a pre-PCR treatment. However, slight mismatches are possible as the first five cycles are less stringent, and not all markers may linked to the gene of interest (Palumbo et al., 2007).

Applications: TRAP has been applied in mapping and tagging disease resistance traits in common beans., assessing the genetic diversity among sugarcane germplasm collections, and for marker-trait association studies (Alwala et al., 2006; Kumar et al., 2014; Miklas et al., 2006).

#### 5.1.5 Conserved region amplification polymorphism (CoRAP)

Background: CoRAP markers have been developed by combining principles of “SRAP” and “TRAP.” The method relies on a two-primer PCR amplification assay, with one arbitrary and one fixed primer. The fixed primer, similar to TRAP, is derived from the EST sequence of the target candidate genes, and the arbitrary primer is similar to the SRAP markers. It contains a conserved intron-specific sequence (CACGC). While the fixed primer provides specificity, the arbitrary primer promotes genome-wide accessibility (Wang et al., 2009). CoRAP are dominant markers and are locus-specific. The variation within the individual genomes leads to polymorphism, which can visualised on a polyacrylamide gel.

Pros and cons: CoRAP uses two primers, one derived from EST, which provides gene specificity. The other arbitrary primer ensures genome-wide accessibility and is more reliable than TRAP, easy to use, and reproducible) (Fabriki-Ourang and Yousefi-Azarkhanian, 2018; Wang et al., 2009).

Applications: CoRAP has been used to study the genetic relationship among 25 Salvia ecotypes/species using TRAP and CoRAP markers. Using a combination of 12 primers (four pairs of arbitrary primers and three pairs of fixed primers derived from EST sequences of *Salvia miltorrhiza)*, 180 polymorphic loci (100%) data were obtained from this analysis, which helped the authors in clustering the 25 ecotypes/species into five major groups (Fabriki-Ourang and Yousefi-Azarkhanian, 2018).

#### 5.1.6 Cytochrome P450-based analogs (PBA)

Background: The PBA markers originate from the cytochrome P450 analogues, a monooxygenase enzyme conserved from animals to microbes, including plants. The enzyme plays a vital role in the biosynthesis of secondary metabolites and oxidative detoxification in plants, and it has a catabolic role in bacteria and oxidation of fatty acids and drug metabolism in animals (Behrendorff, 2021; Guengerich, 2021; Yamanaka et al., 2003). Yamanaka et al. (2003) developed these markers using PCR primers of mammalian P450 analogues in plants and used them to analyse intra and interspecific diversity within 51 plant species of 28 taxonomic families. The polymorphism can be observed on an agarose gel (Yamanaka et al., 2003).

Pros and cons: These functionally conserved genes are highly diverse in their genomic distribution. These are simple to use as universal primers exist. However, they may have PCR-related issues, resulting in technical failures (Poczai et al., 2013).

Application: Saini et al. (2013) used PBA markers to assess *Moringa oleifera* lam’s genetic variability. Seven pairs of PBA primers were used, and 40% polymorphism was identified. However, 48.68% and 48.57% polymorphism were reported for RAPD and ISSR primers, respectively. This study provides the practical applications of the PBA markers, where their role in genetic diversity and systematic breeding has been mentioned (Saini et al., 2013).

#### 5.1.7 Tubulin-based polymorphism (TBP)

Background: TBP are markers based on the polymorphism within the introns of β-tubulins. Utilising the ubiquity of this gene, the primers designed for the first intron of different β-tubulin isotypes can reveal diverse fingerprints. The principle lies in the design of primers, targeting the difference in intron length. The polymorphism can be visualised on an agarose gel (Bardini et al., 2004). These are co-dominant markers but are not locus-specific (Morello et al., 2019).

Pros and cons: The method is simple and user-friendly, does not require prior sequence information for assay, and allows DNA barcoding. The intron is flanked by coding sequences on either side, enabling a more straightforward primer design. These are helpful when prior sequence information is unavailable (Bardini et al., 2004).

Applications: The markers have been applied to various plant species, such as *Triticum*, *Camelina sativa,* and grapes (Galasso et al., 2011; Gavazzi et al., 2016; Guadalupi et al., 2022). Gavazzi et al. (2016) used TBP as an alternative tool for genetically profiling grapes besides SSR. They could deduce comparable TBP results to six internationally recognised SSR markers (Gavazzi et al., 2016). Gianì et al. (2020) generated a genomic profile using TBP to authenticate animal species in meat and poultry. The study observed that each species has a distinct diagnostic fragment, which can assist in species-specific identification (Gianì et al., 2020).

### 5.2 Non-coding-based targeted method for genotyping

Non-gene-based functional markers are not easy to identify because these are located in the intergenic and noncoding regions of the genome. However, these markers may be significantly associated with or involved in regulating the trait of interest. The fundamental outline of non-coding marker development is similar to the gene-targeted functional marker discovery, except for targeting non-coding DNA sequences (SNPs, repetitive sequences, consensus sequences) or differentially expressed non-coding molecules (non-coding RNA elements). A detailed discussion on identifying these elements is beyond the current scope of this article. The bioinformatic resources valuable in the identification of essential non-coding elements are databases such as RNAcentral, Rfam, UCNE, Repbase, SINEBase; pipelines such as Transcriptator, Sebnif, etc., for noncoding RNA element discovery; Piler, RepeatExplorer and RepeatMasker for repetitive DNA discovery (Dimitrieva and Bucher, 2013; Griffiths-Jones et al., 2003; Huang et al., 2016; Petrov et al., 2017; Sun et al., 2014; Tripathi et al., 2015).

#### 5.2.1 Intron-targeted amplified polymorphism (ITAP)

Background: ITAP markers are based on polymorphism within the intron sequences (Poczai et al., 2013), such as using intron-exon splice junction targeted primers in identifying polymorphisms and their role in mapping polymorphic sites for various studies (Weining and Langridge, 1991). Xiong et al. (2013) developed ITAP based on the principles of arbitrary and EST-derived intron-exon splice junction primers. The method uses two primers, one targeting the intron-exon splice junction, the other derived as SRAP markers. ITAP are dominant markers, but no information is reported for loci specificity.

Pros and cons: Introns are sparsely distributed throughout the genome, and more events of genomic rearrangements occur within the introns than exons, a better indicator of polymorphism. The ITAP protocol is relatively simple and inexpensive and has been used for various applications.

Applications: ITAP markers have been used for various applications like genetic diversity assessment and sex identification of date palms and analysing the introgression of iron and zinc transfer-specific genes in *Aegilops* species (Atia et al., 2017a; Atia et al., 2017b; Xiong et al., 2013).

#### 5.2.2 Inter-SINE amplified polymorphism (ISAP) system

Background: A group of retro-transposons called short interspersed nuclear elements (SINEs) are short, repetitive, non-coding sequences, about 100–600 bp. SINEs are widely distributed throughout the genome and have various roles, such as genome organisation, evolution, and gene expression modulation (Kanhayuwa and Coutts, 2016). Therefore, these can be used as markers; the principle behind the method lies in the outward-facing primers that target the highly conserved SINE sequences. The variation within the amplicon product enables the polymorphism studies. These are dominant markers and locus-specific (Reiche et al., 2021; Seibt et al., 2012).

Pros and cons: ISAP markers are robust, cheaper than other markers such as AFLP, and can be easily automated through capillary electrophoresis. They do not require extensive laboratory equipment and can be analysed on an agarose gel. ISAP markers are more reproducible than RAPD, which provide similar informative banding. The directionality of change in ISAP can be determined, which cannot be done for SNP markers (Reiche et al., 2021; Seibt et al., 2012). ISAP marker development requires knowledge of the SINE sequences of the target species, and the variation among species is a significant limitation for wide-scale application.

Application: ISAP markers were developed to genotype potatoes; however, they have been translated for application in many other plant species. Sormin et al. used ISAP markers to genotype melon (*Cucumis melo).* The study includes the identification of melon-specific SINEs, and these melon-specific ISAP markers showed a higher polymorphism than potato-specific markers. The melon-specific ISAP markers were also tested in *Coleus* species to test their broader application. It was possible to differentiate between different *Coleus* accessions using melon-specific ISAPs (Sormin et al., 2021).

#### 5.2.3 Sequence-characterised amplified region (SCAR)

Background: SCAR is a genomic DNA fragment derived from a single locus identified by a pair of specific oligonucleotide primers. Paran and Michelmore first developed the markers in the 1990 s by cloning and sequencing locus-specific amplicons of RAPD markers. They designed 24-mer oligonucleotide primers using the sequence information from the single loci clone (Paran and Michelmore, 1993). SCAR is primarily a co-dominant locus-specific marker.

Pros and cons: SCAR overcomes the limitations of RAPD markers of reaction sensitivity and is more reliable than RAPD. The SCAR markers are specific to particular loci and can help develop specific markers for diagnostic or genotyping purposes (Amicucci et al., 1997; Paran and Michelmore, 1993). The only disadvantage of the technique is the laborious procedure. It is essential to clone and sequence the specific loci to design these primers.

Application: Yadav et al. (2012) developed SCAR markers to identify *Bacopa monnieri* from adulterants like *Centella asiatica*, *Eclipta alba*, and *Malva rotundifolia*. It could be cloned and sequenced using multiple RAPD primers and identifying plant-specific bands, and specific SCAR markers for *Bacopa monnieri* could be designed (Yadav et al., 2012). In another study, Didion et al. (2001) mapped a SCAR sequence to chromosome 6 from Porcine DNA, a fragment of 255 kb loci called DK122 (Didion et al., 2001).

#### 5.2.4 Sequence-specific amplified polymorphism (SSAP)

Background: In Waugh et al. (1997) developed SSAPs to target transposable elements as markers. The SSAP markers are created by designing PCR primers that amplify the region between a priming site near the end of a transposable element and an adjacent restriction site flanking in the genomic DNA (Syed and Flavell, 2006). AFLP markers’ technique inspires the principle of SSAP but differs in the adaptor ligation, where a known sequence of Bare-1 specific radiolabeled primer was used as an adapter. These are dominant, locus-specific markers (Waugh et al., 1997).

Pros and cons: SSAPs are similar to the AFLP markers but identify retrotransposon insertion-mediated polymorphisms. These are highly informative, reliable, and efficient in detecting changes at individual and population levels (Jiang et al., 2016; Li et al., 2009). However, these markers require the genetic information of the retrotransposons of interest, which has limited its wide-scale application. Besides, it is a one-to 2-week process, making it laborious and cumbersome.

Applications: Berenyi et al. (2002) developed SSAP markers for the genetic analysis of sweet potatoes using the *TY-1 copia* retrotransposon. It has also been used in genotyping *Vitis vinifera* by creating SSAP markers for the Vine-1 retrotransposon (Berenyi et al., 2002; Labra et al., 2004).

#### 5.2.5 Inter-small RNA polymorphism (iSNAP)

Background: iSNAPs are small RNA-based molecular markers developed by Gui et al. (2011). They use the presence of small non-coding RNA of 20–24 nucleotides in length across the genome for marker development. The primer design explains these principles, which include mapping multiple small RNAs across the genome and designing primers at the two flanking ends of these multi-mapped small RNAs. The primers can be created within the small RNAs or their conserved 5′ and 3′ flanking regions (Gui et al., 2011).

Pros and cons: While the markers are reliable, offer high throughput, and are based on non-coding sequences (Poczai et al., 2013), they involve complex procedures and can be expensive regarding the sequencing costs associated with marker development.

Application: Other than the original study, no application studies were reported in the public domain. However, a few studies on related approaches which exploit miRNAs and other small RNA molecules in plant functional studies have been cited (Htwe et al., 2015; Solofoharivelo et al., 2014).

#### 5.2.6 Promoter-anchored amplified polymorphism (PAAP)

Background: PAAP are markers developed by Pang et al. (2009) using conserved promoter sequences of the cotton genome. They combined an RAPD primer and a degenerate promoter-specific primer to identify the polymorphism in the cotton genome and visualised it on a polyacrylamide gel (Pang et al., 2009).

Pros and cons: These are more reliable and informative than RAPD primers. However, they require the promoter information of the gene of interest, which may only be available for some species.

Applications: Mokate et al. (2017) used PAAP to assess divergence in *Glycine max* (Soybean). They observed that these markers produced significant polymorphic loci and highlighted the application of PAAP to study particular traits (Mokate et al., 2017). Table 2 lists the principles and PCR strategy for commonly used functional markers and their targets.

**TABLE 2:** A summary of targeted and non-targeted functional DNA markers, their targets and used PCR strategies.

Marker	Target	PCR strategy	Detection
SCOT	Start codon	Single primer-based amplification assay targeting the start codon	AGE
CDDP	Conserved DNA regions of specific genes	Single primer-based amplification assay targets conserved DNA priming sites with differential distribution across the genome	AGE
SRAP	Open Reading frame	It uses two primers with a distinct primer structure, a random filler sequence, an AT OR GC-rich core, and 3 selective nucleotides at the 3′end to enable the amplification of various regions of the genome	PAGE
TRAP	EST	It amplifies various genome regions using an arbitrary SRAP primer and an EST sequence-derived primer	PAGE
CoRAP	Conserved intron/EST	It uses two primers: a fixed primer from EST sequences, an arbitrary primer with a core sequence ‘CACGC,’ a plant-specific intron sequence	PAGE
PBA	Cytochrome P450-based analogue sequences	Universal primers targeting the P450 genes	AGE
TBP	Β-Tubulim	A single degenerate primer binds to the conserved introns of the β-tubulin. Polymorphism is based on varied intron length	AGE
ITAP	Intron/exon splice junctions	In two primers-based amplification assays, one primer is derived from SRAP, and the other targets the intron/exon splice junction	AGE
ISAP	SINES	Two primers-based amplification assays, designed with SINE elements to amplify the adjacent genomic regions	AGE
SCAR	Sequence-Specific clones	Two primer-based amplification assays, with the primers derived from single loci clones from unique RAPD, AFLP bands	AGE
SSAP	Transposable elements	Restriction digestion was followed by two primer amplifications, one LTR-specific and the other adapter-specific	PAGE
iSNAP	Small RNAs	Two primer amplification assays, where primers are designed within and at the flanking regions of small RNAs mapped on the genome	PAGE
PAAP	Promoters	Two primer-based amplification assays, with a plant-promoter-specific primer and an RAPD primer	PAGE

AGE, agarose gel electrophoresis; PAGE, polyacrylamide gel electrophoresis.

#### 5.2.7 Single nucleotide polymorphism (SNP) markers

SNP is a single nucleotide substitution in genome sequences (Schork et al., 2000). Genome-wide distribution and automation amenability have made them the most preferred markers for humans, animals and plant genotyping studies (Zeng et al., 2017). SNPs have revolutionised marker-assisted studies in humans, animals and plants (Gao et al., 2023; Li et al., 2023; Pavlova et al., 2023; Raza et al., 2023). At the same time, there are many different ways of SNP genotyping, such as PCR, restriction digestion associated DNA sequencing, pyrosequencing and SNP arrays (Lawrie and Massey, 2023). SNP arrays are most popular, Various SNP arrays have been made for many organisms, including humans (Affymetrix Human SNP Array 6.0, Illumina HumanOmniZhongHua-8 Beadchip), dogs (Affymetrix Canine SNP Genotyping Array, Illumina CanineHD Beadchip), and cattle (Affymetrix Genome-Wide BOS 1 Bovine Array, Illumina BovineHD BeadChip) (Liu et al., 2017). A large number of GWAS studies have been conducted using these SNP chips. SNPs are widely used in various domains of biological sciences, which could be difficult to accommodate here. Therefore, a brief description of the different SNP arrays and their application in humans, animals, and plants is provided in the Supplementary Material.

#### 5.2.8 Repetitive DNA markers

Repetitive DNA sequences are the homologous DNA sequences found in multiple genome copies. Their genome-wide occurrence makes them a potential marker. They can be grouped based on occurrences such as highly repetitive (Satellite DNA) and moderately repetitive DNA sequences (Tandem and interspersed repeat sequences) (Pelley, 2007). DNA tandem repeats can be further categorised into microsatellites, minisatellites, and megasatellites (Trent, 2012), whereas interspersed repeat sequences are further classified into RNA and DNA transposons-based markers. Repetitive DNA markers offer the advantages of co-dominance and genome-wide polymorphism coverage. It is widely used in plant and animal genotyping studies (Bora et al., 2023; Lahkar et al., 2023; Liu et al., 2023; Loukovitis et al., 2023; Mauger et al., 2023; Yeo et al., 2023). The scope of the current review is limited to the random and functional markers. Therefore, only a brief description of the marker and the application of each repetitive DNA marker is provided in the Supplementary Material.

## 6 Selection of a marker for genotyping

**FIGURE 5:**
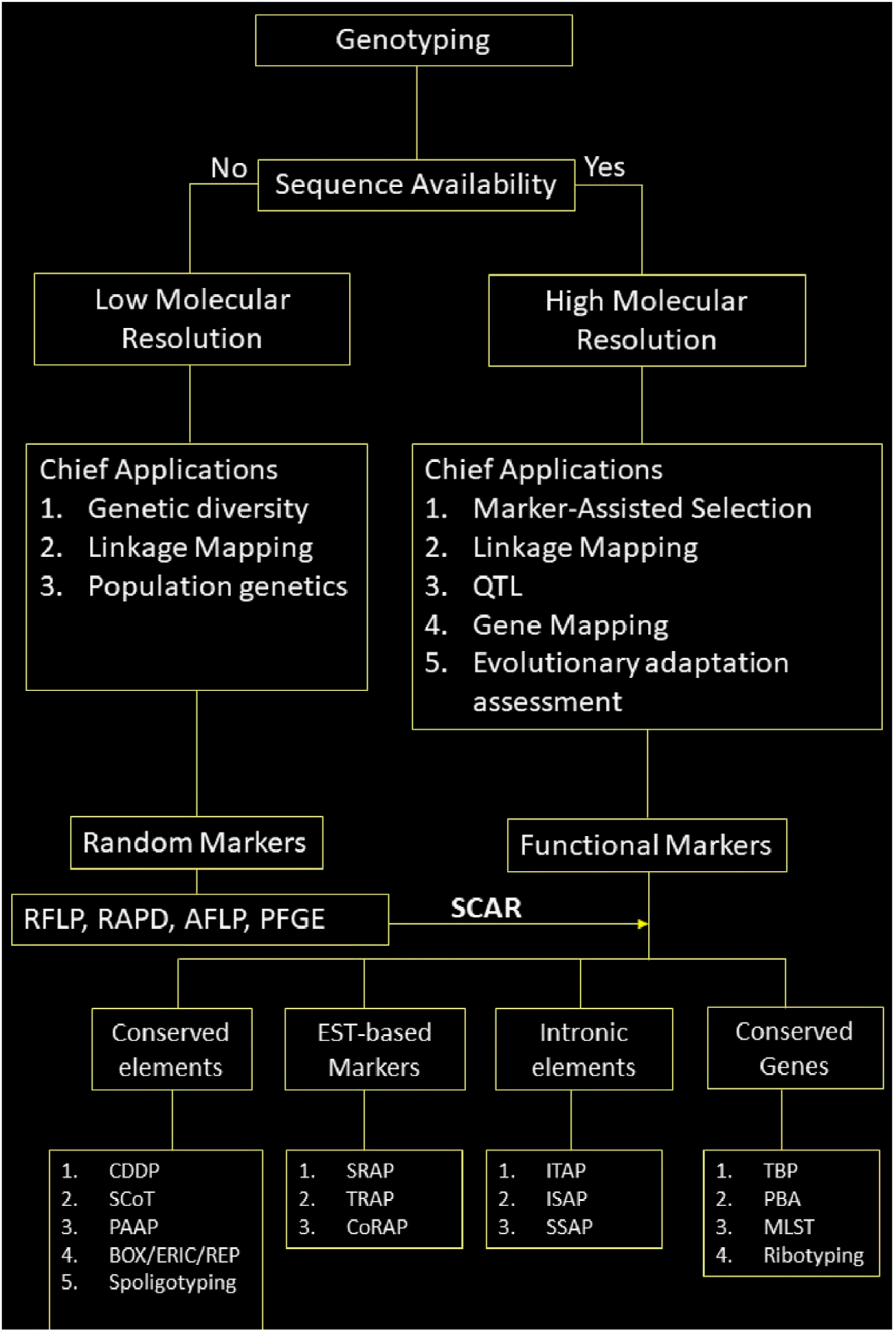
Schematic representation of marker selection for genotyping; note: Only chief applications of the markers based on their properties has been given here, they may be used for other applications as well.

The plethora of DNA markers has made it easier to genotype organisms for various applications. However, not all the markers are suitable for all the applications. There is no one-size-fits-all solution for the genotyping problem. As discussed above, selecting a marker depends upon various factors, such as the study’s objective, budget, resources, and specific applications. To facilitate the choice of marker, we schematically represent a workflow that aids in marker selection based on the availability of genome sequence, its chief applications, and the elements it targets within the genome in Figure 5.

## 7 Conclusion

Understanding the genetic variation between species or populations is essential to knowing how different groups have evolved and diverged with genomic merits. Genotyping has quintessential roles in revealing the genetic diversity among populations and in genetics research. Therefore, various genotyping markers have been developed and are highly used for their simplicity, low cost, and ease of handling. However, these are also packed with specificity, reproducibility, and large sample handling issues. While random markers are outdated, their application continues in livestock with some modifications. The advent of genome sequencing and bioinformatics tools facilitated the development of functional markers to overcome their limitations, showing high specificity and direct linkage with phenotypic traits. However, the application of functional markers is mainly limited to plants and remains underutilised in livestock. This can be reasoned by the animals’ relatively less studied complex genome, lack of bioinformatics resources, and the complexity of designing these markers. Despite these, functional markers have massive potential beyond the genomic diversity and breeding investigations in animal sciences. High-throughput markers like SNP have overtaken their application in livestock, which are expensive and require high technical expertise and logistics. Gene-specific functional markers are handy, considering the cost of various marker-assisted breeding applications. Further, the advent of multi-omics and bioinformatics tools and techniques in livestock breeding can accelerate functional marker discovery and subsequent low-cost validation.

## 8 Future perspectives

The underutilised potential of functional markers and the limitations of random markers invite many innovations and offer a fresh perspective towards applying genetic markers in livestock research.

• RFLP markers are highly reproducible and co-dominant, but their low throughput per sample limits them. Extending automated RFLP markers to the high-throughput platform can be an excellent solution.
• RAPD markers are notorious for their lack of reproducibility, mainly due to their arbitrary nature. The problem is partially addressed by SCoT and CDDP markers, but developing SCAR markers from locus-specific RAPD markers is the most appropriate solution. It is specific to the locus, can be converted into co-dominant markers, and can be automated.
• While AFLP markers overcome the limitations of RFLP and RAPD, they lack co-dominant inheritance expression. This can be very well addressed by locus-specific fragment isolation and subsequent sequence-specific primer designing, i.e., designing SCAR markers.
• The availability of sequence information has accelerated the development of gene-specific functional markers in plant science. Despite the availability of various genomics databases and bioinformatics resources for livestock animals, minimal functional markers are known for genotyping. Reducing sequencing costs and the availability of bioinformatics resources can be exploited to develop functional markers for livestock species as low-cost alternative genotyping markers.
• It is noteworthy that the non-coding DNA is the major polymorphic region. Therefore, a deeper insight into the region can enable the development of markers based on these variations.
• Non-coding elements such as the promoter, LINES, SINES, small RNAs and intergenic regions harbour the majority of regulatory sites in DNA; combined with the power of machine learning tools and algorithms, the marker discovery and its application can be improved in livestock and other agronomically essential species.
• Random and functional markers can be used to develop higher-throughput genotyping methods.
