# Supplementary material for: Genetic marker: a genome mapping tool to decode genetic diversity of livestock animals
Source: Front Genet. 2024 Oct 17;15:1463474. doi: 10.3389/fgene.2024.1463474 (PMC11524813; doi:10.3389/fgene.2024.1463474)
Supplement: Supplementary file 1 [file Presentation1.PPTX]

## Slide 1
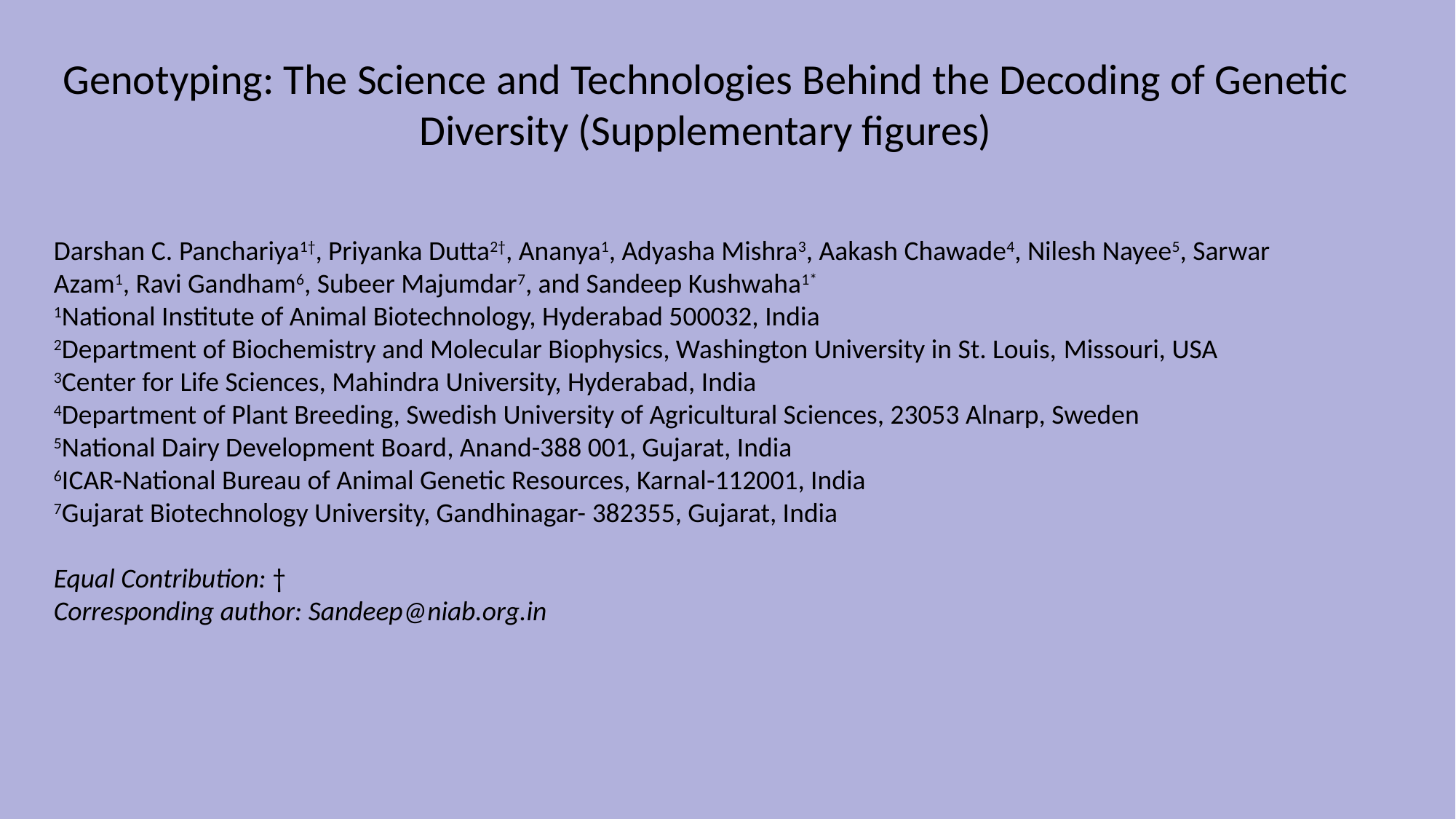

Genotyping: The Science and Technologies Behind the Decoding of Genetic Diversity (Supplementary figures)
Darshan C. Panchariya1†, Priyanka Dutta2†, Ananya1, Adyasha Mishra3, Aakash Chawade4, Nilesh Nayee5, Sarwar Azam1, Ravi Gandham6, Subeer Majumdar7, and Sandeep Kushwaha1*
1National Institute of Animal Biotechnology, Hyderabad 500032, India
2Department of Biochemistry and Molecular Biophysics, Washington University in St. Louis, Missouri, USA
3Center for Life Sciences, Mahindra University, Hyderabad, India
4Department of Plant Breeding, Swedish University of Agricultural Sciences, 23053 Alnarp, Sweden
5National Dairy Development Board, Anand-388 001, Gujarat, India
6ICAR-National Bureau of Animal Genetic Resources, Karnal-112001, India
7Gujarat Biotechnology University, Gandhinagar- 382355, Gujarat, India
Equal Contribution: †
Corresponding author: Sandeep@niab.org.in

## Slide 2
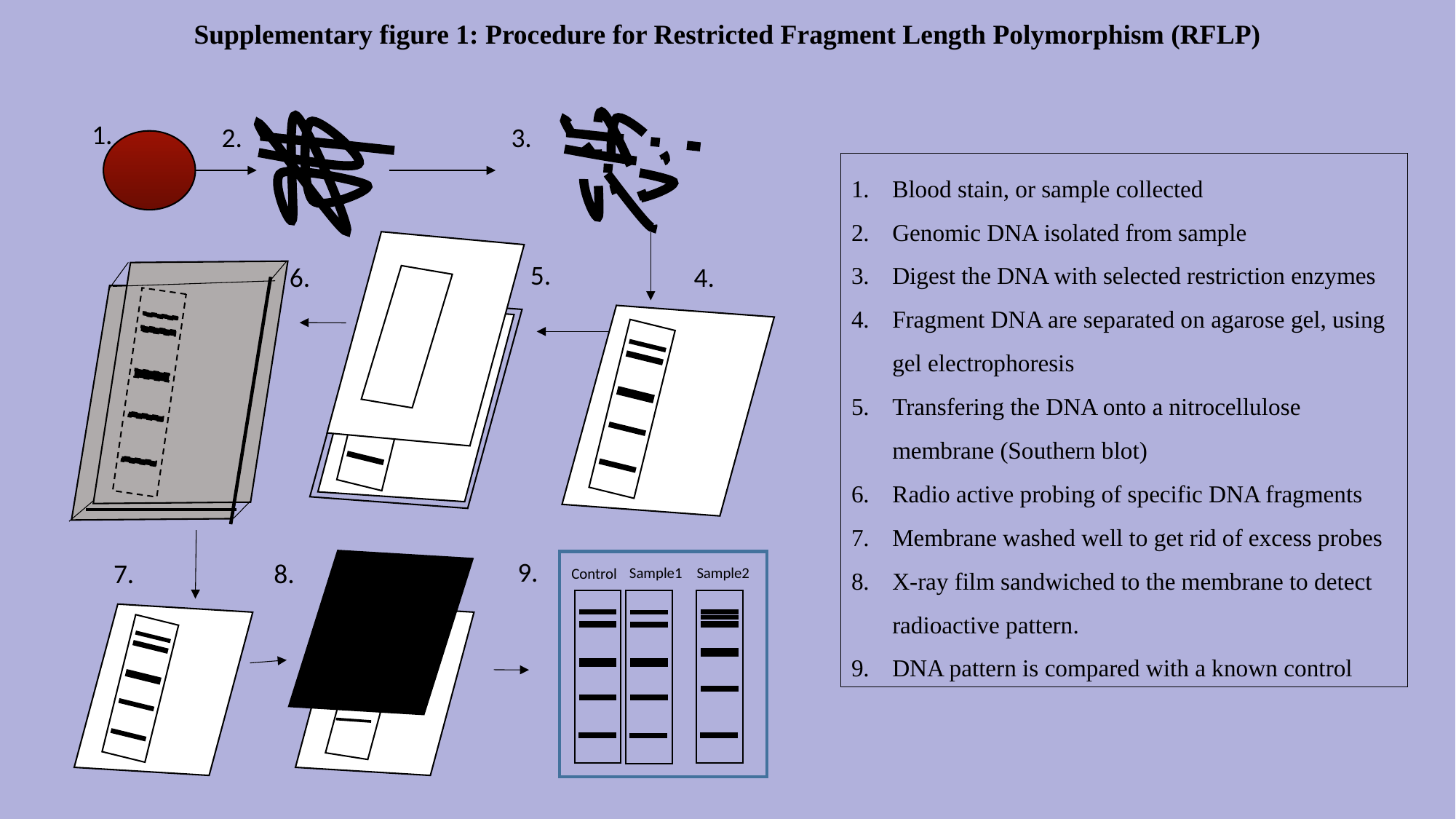

Supplementary figure 1: Procedure for Restricted Fragment Length Polymorphism (RFLP)
1.
2.
3.
5.
6.
4.
9.
8.
7.
Sample2
Sample1
Control
Blood stain, or sample collected
Genomic DNA isolated from sample
Digest the DNA with selected restriction enzymes
Fragment DNA are separated on agarose gel, using gel electrophoresis
Transfering the DNA onto a nitrocellulose membrane (Southern blot)
Radio active probing of specific DNA fragments
Membrane washed well to get rid of excess probes
X-ray film sandwiched to the membrane to detect radioactive pattern.
DNA pattern is compared with a known control

## Slide 3
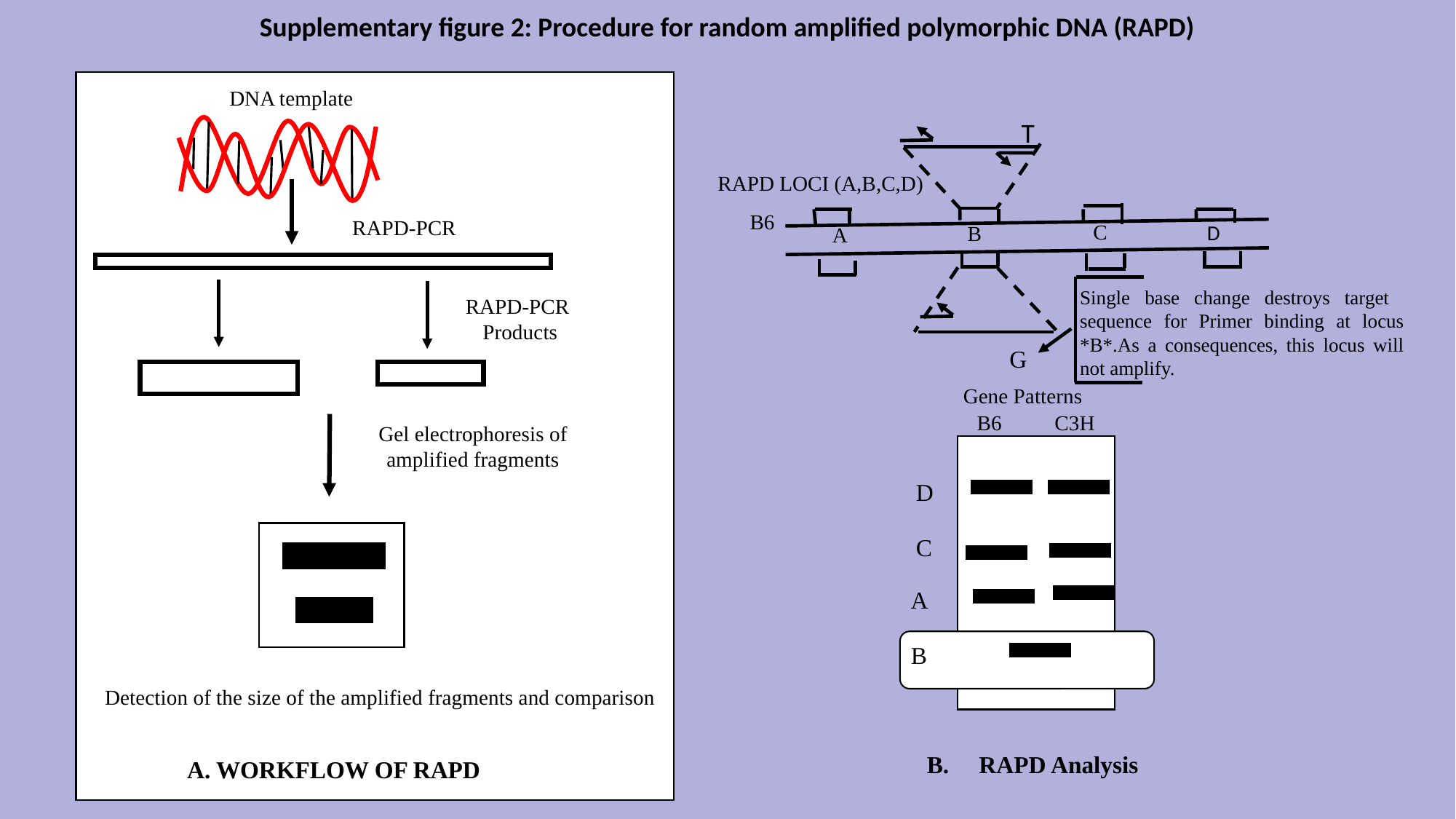

Supplementary figure 2: Procedure for random amplified polymorphic DNA (RAPD)
DNA template
T
RAPD LOCI (A,B,C,D)
B6
RAPD-PCR
C
D
B
A
Single base change destroys target sequence for Primer binding at locus *B*.As a consequences, this locus will not amplify.
RAPD-PCR Products
G
Gene Patterns
 B6 C3H
Gel electrophoresis of amplified fragments
D
C
A
B
Detection of the size of the amplified fragments and comparison
B. RAPD Analysis
A. WORKFLOW OF RAPD

## Slide 4
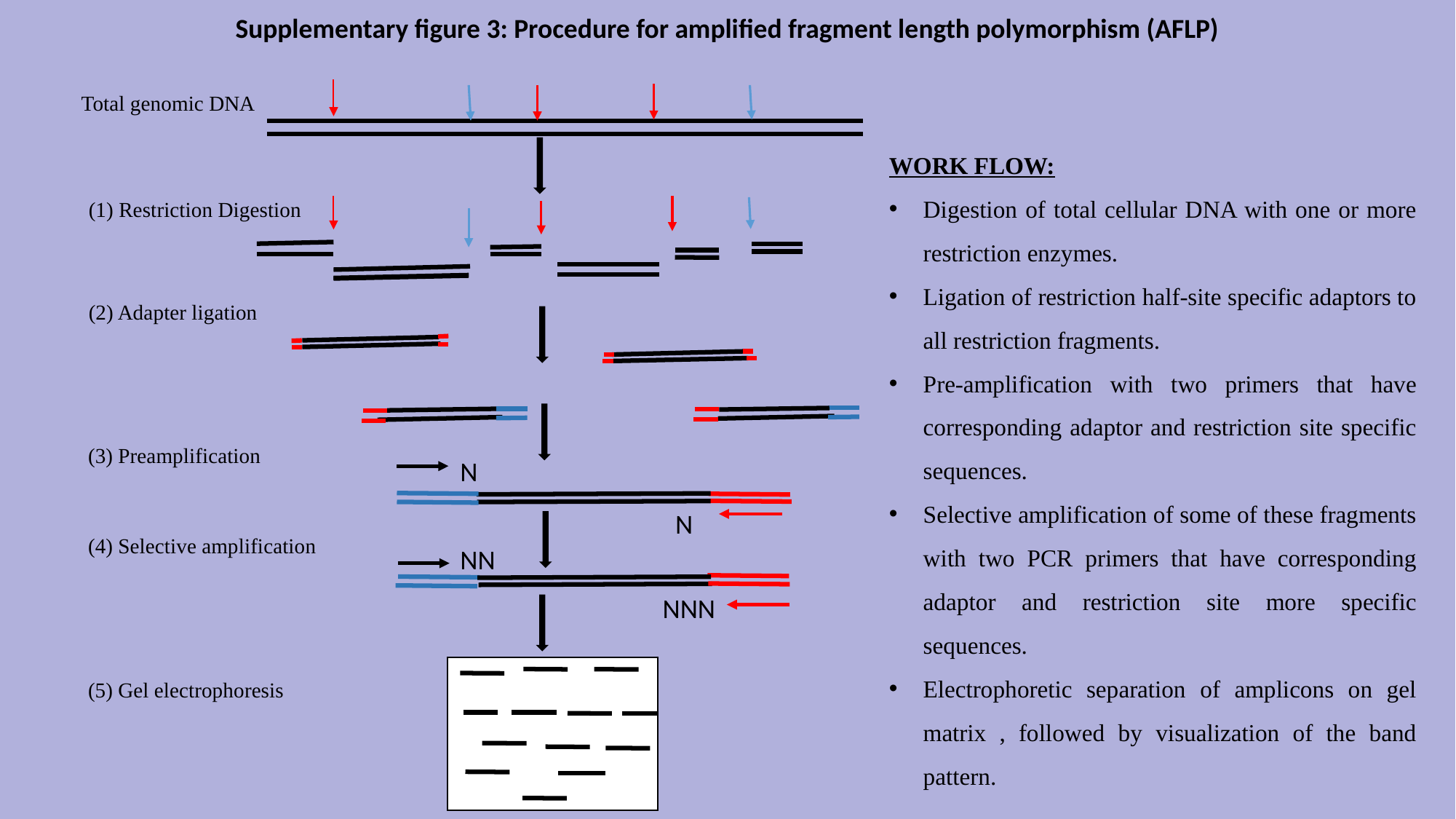

Supplementary figure 3: Procedure for amplified fragment length polymorphism (AFLP)
Total genomic DNA
WORK FLOW:
Digestion of total cellular DNA with one or more restriction enzymes.
Ligation of restriction half-site specific adaptors to all restriction fragments.
Pre-amplification with two primers that have corresponding adaptor and restriction site specific sequences.
Selective amplification of some of these fragments with two PCR primers that have corresponding adaptor and restriction site more specific sequences.
Electrophoretic separation of amplicons on gel matrix , followed by visualization of the band pattern.
(1) Restriction Digestion
(2) Adapter ligation
(3) Preamplification
N
N
(4) Selective amplification
NN
NNN
(5) Gel electrophoresis

## Slide 5
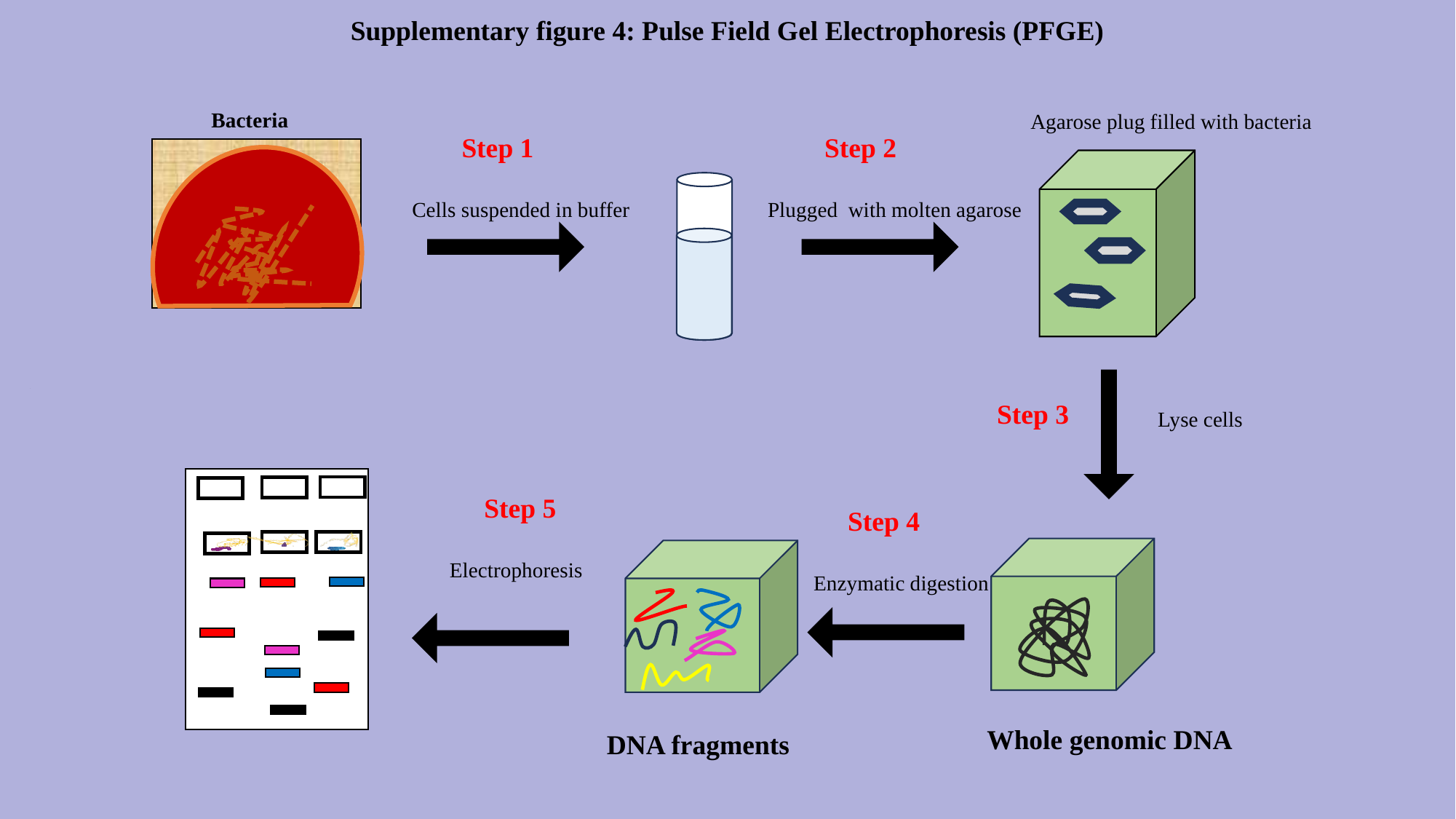

Supplementary figure 4: Pulse Field Gel Electrophoresis (PFGE)
Bacteria
Agarose plug filled with bacteria
 Step 2
 Plugged with molten agarose
 Step 1
 Cells suspended in buffer
Step 3
Lyse cells
 Step 5
Electrophoresis
 Step 4
 Enzymatic digestion
Whole genomic DNA
DNA fragments

## Slide 6
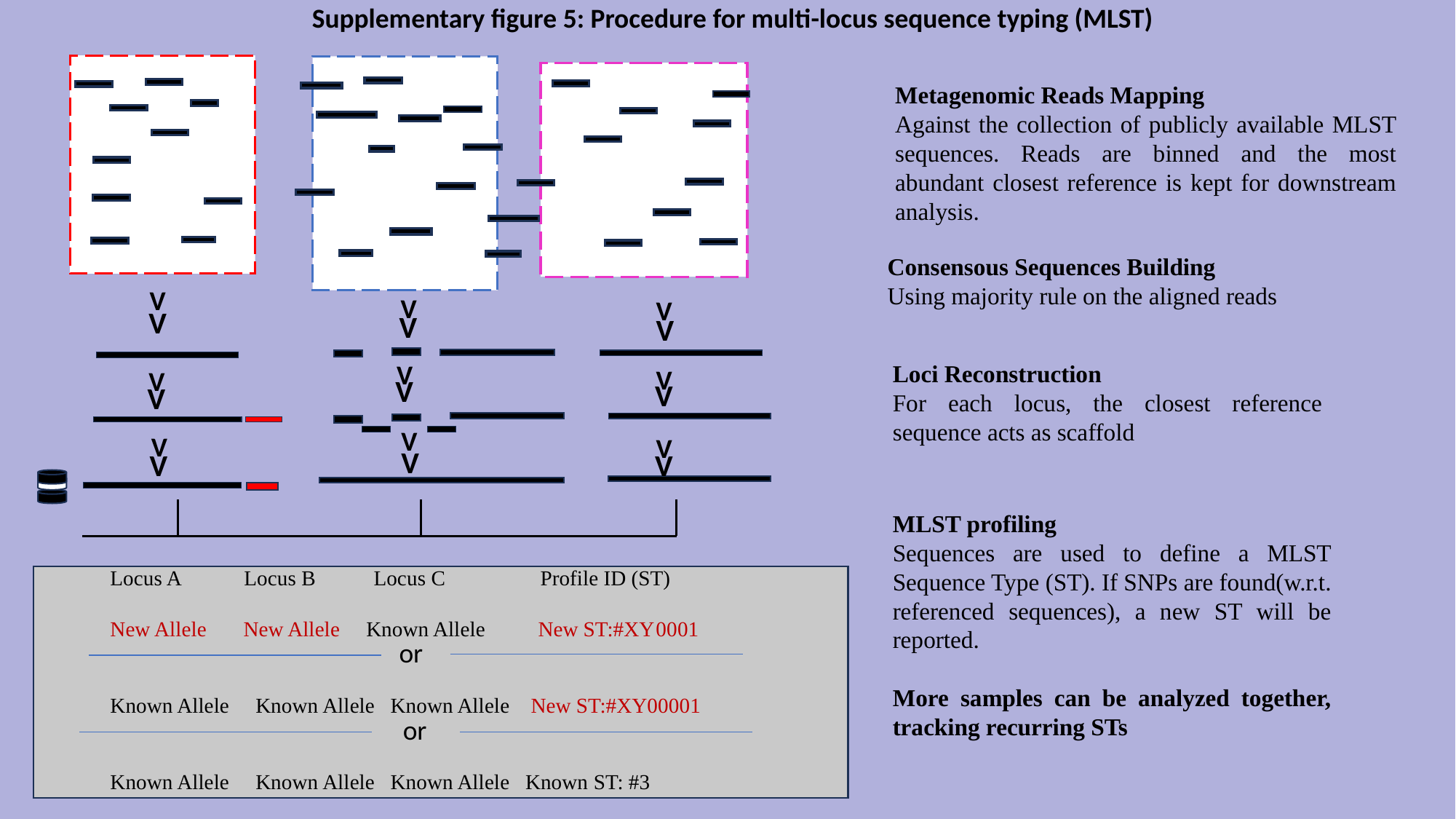

Supplementary figure 5: Procedure for multi-locus sequence typing (MLST)
Metagenomic Reads Mapping
Against the collection of publicly available MLST sequences. Reads are binned and the most abundant closest reference is kept for downstream analysis.
Consensous Sequences Building
Using majority rule on the aligned reads
V
V
V
V
V
V
V
Loci Reconstruction
For each locus, the closest reference sequence acts as scaffold
V
V
V
V
V
V
V
V
V
V
V
MLST profiling
Sequences are used to define a MLST Sequence Type (ST). If SNPs are found(w.r.t. referenced sequences), a new ST will be reported.
More samples can be analyzed together, tracking recurring STs
Locus A Locus B Locus C Profile ID (ST)
New Allele New Allele Known Allele New ST:#XY	0001
Known Allele Known Allele Known Allele New ST:#XY00001
Known Allele Known Allele Known Allele Known ST: #3
or
or

## Slide 7
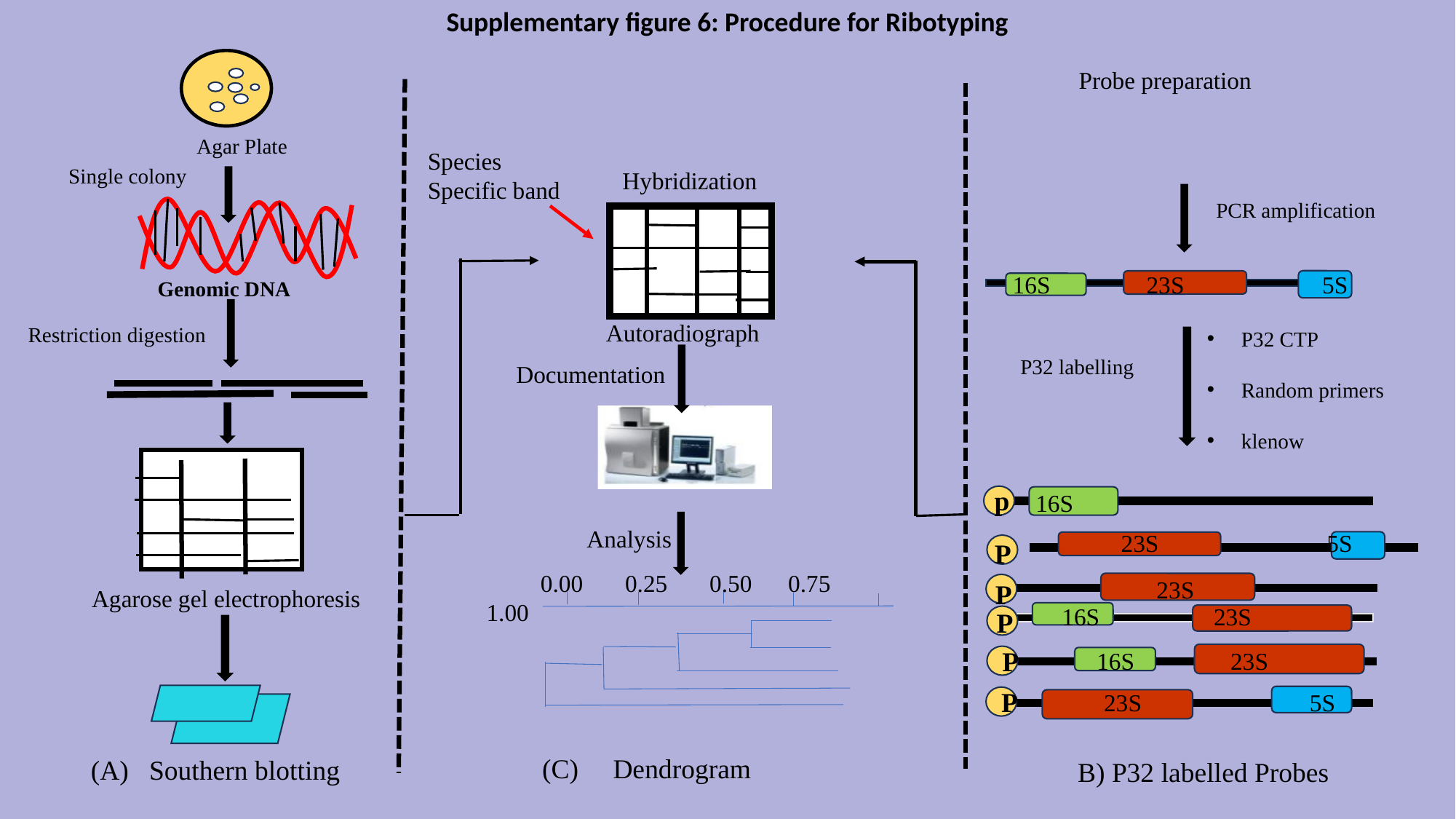

Supplementary figure 6: Procedure for Ribotyping
Probe preparation
Agar Plate
Species
Specific band
Single colony
Hybridization
PCR amplification
16S 23S 5S
Genomic DNA
Autoradiograph
Restriction digestion
P32 CTP
Random primers
klenow
P32 labelling
Documentation
p
16S
Analysis
23S 5S
P
 0.00 0.25 0.50 0.75 1.00
23S
P
Agarose gel electrophoresis
16S 23S
P
16S 23S
P
 23S 5S
P
 (C) Dendrogram
 (A) Southern blotting
B) P32 labelled Probes

## Slide 8
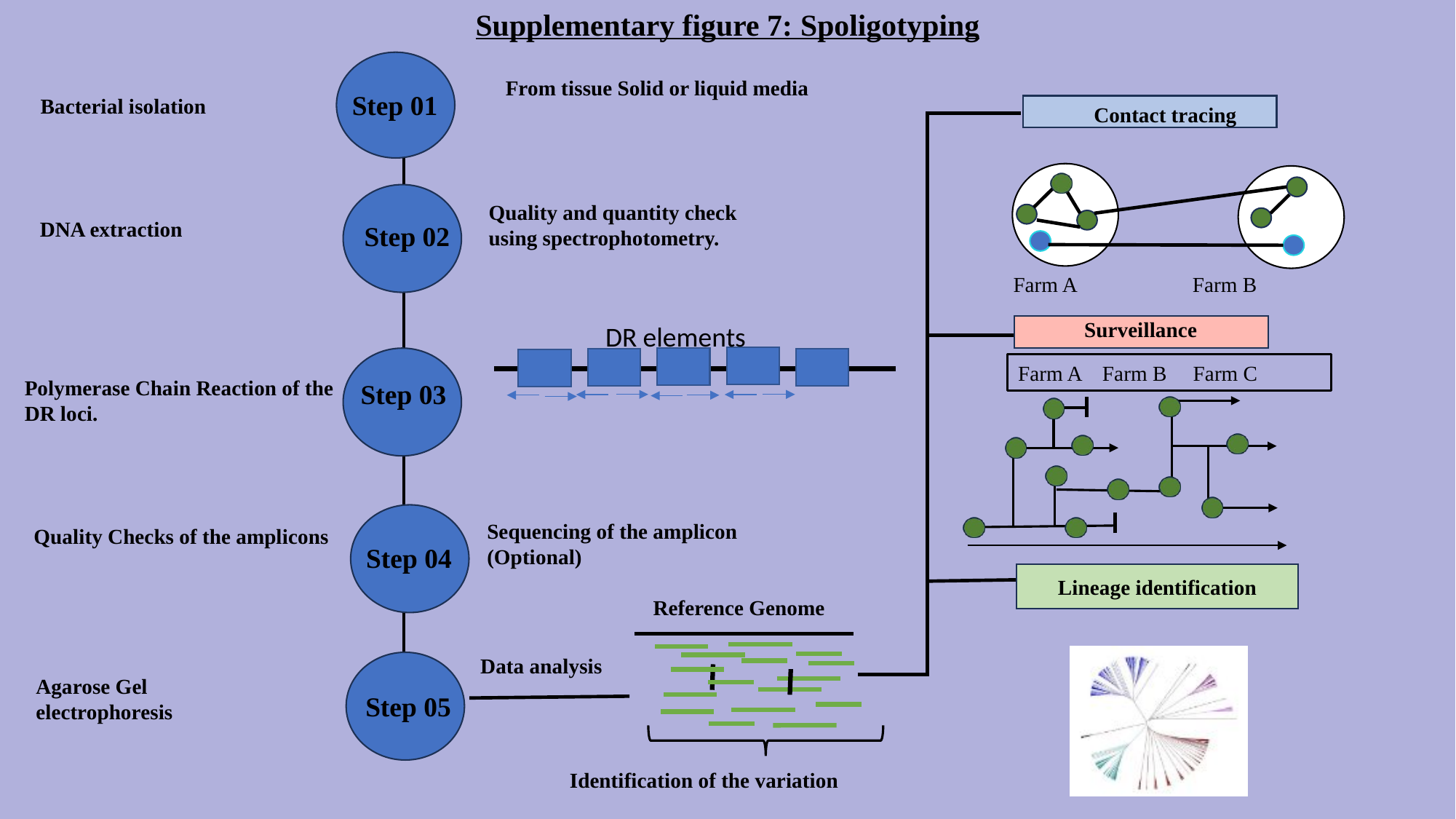

Supplementary figure 7: Spoligotyping
Contact tracing
Surveillance
Lineage identification
Step 01
Bacterial isolation
Quality and quantity check using spectrophotometry.
DNA extraction
Farm A Farm B
Farm A Farm B Farm C
Polymerase Chain Reaction of the DR loci.
Quality Checks of the amplicons
Reference Genome
Data analysis
Agarose Gel electrophoresis
Identification of the variation
From tissue Solid or liquid media
Step 02
DR elements
Step 03
Sequencing of the amplicon (Optional)
Step 04
Step 05

## Slide 9
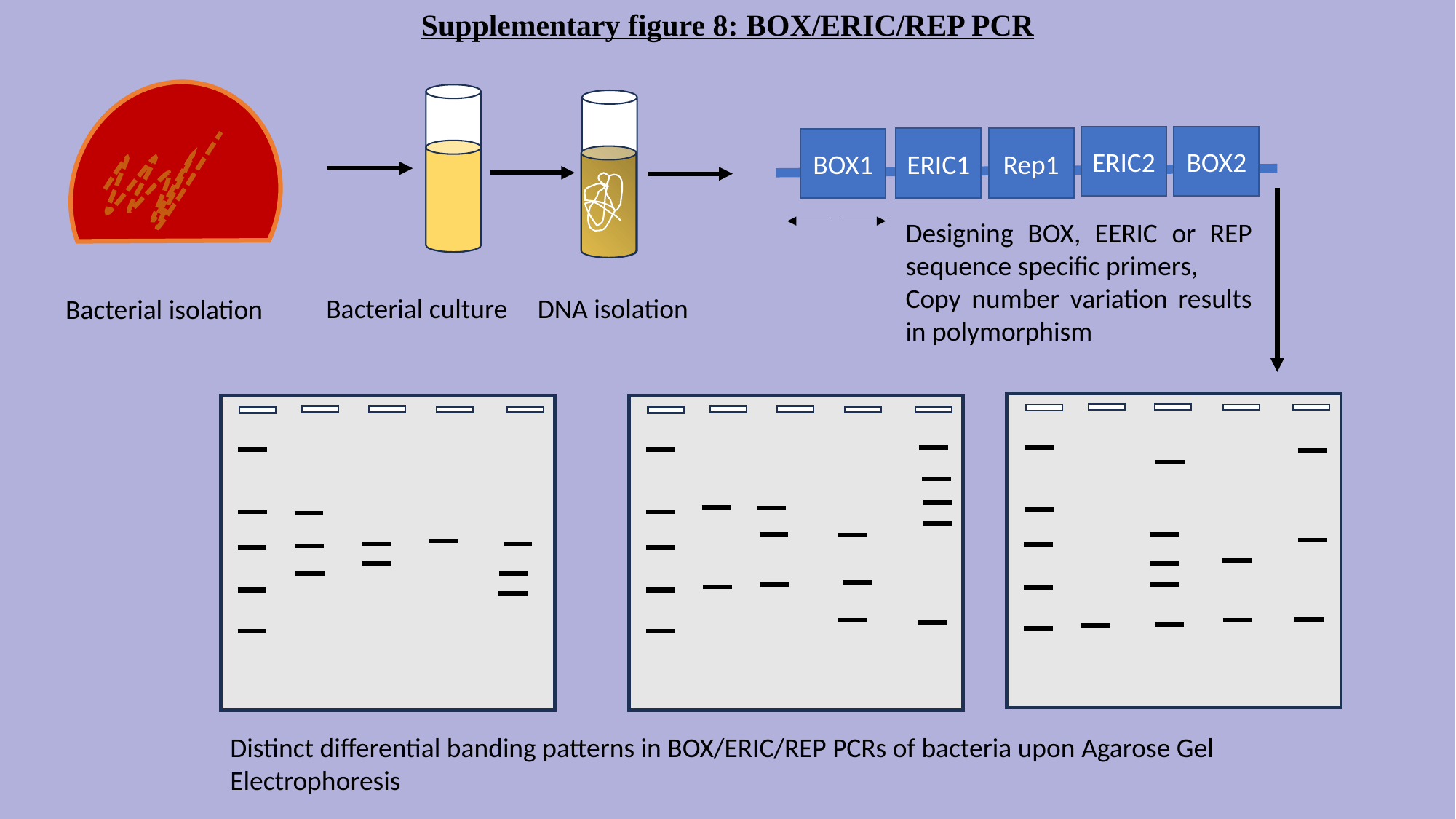

Supplementary figure 8: BOX/ERIC/REP PCR
ERIC2
BOX2
Rep1
ERIC1
BOX1
Designing BOX, EERIC or REP sequence specific primers,
Copy number variation results in polymorphism
DNA isolation
Bacterial culture
Bacterial isolation
Distinct differential banding patterns in BOX/ERIC/REP PCRs of bacteria upon Agarose Gel Electrophoresis

## Slide 10
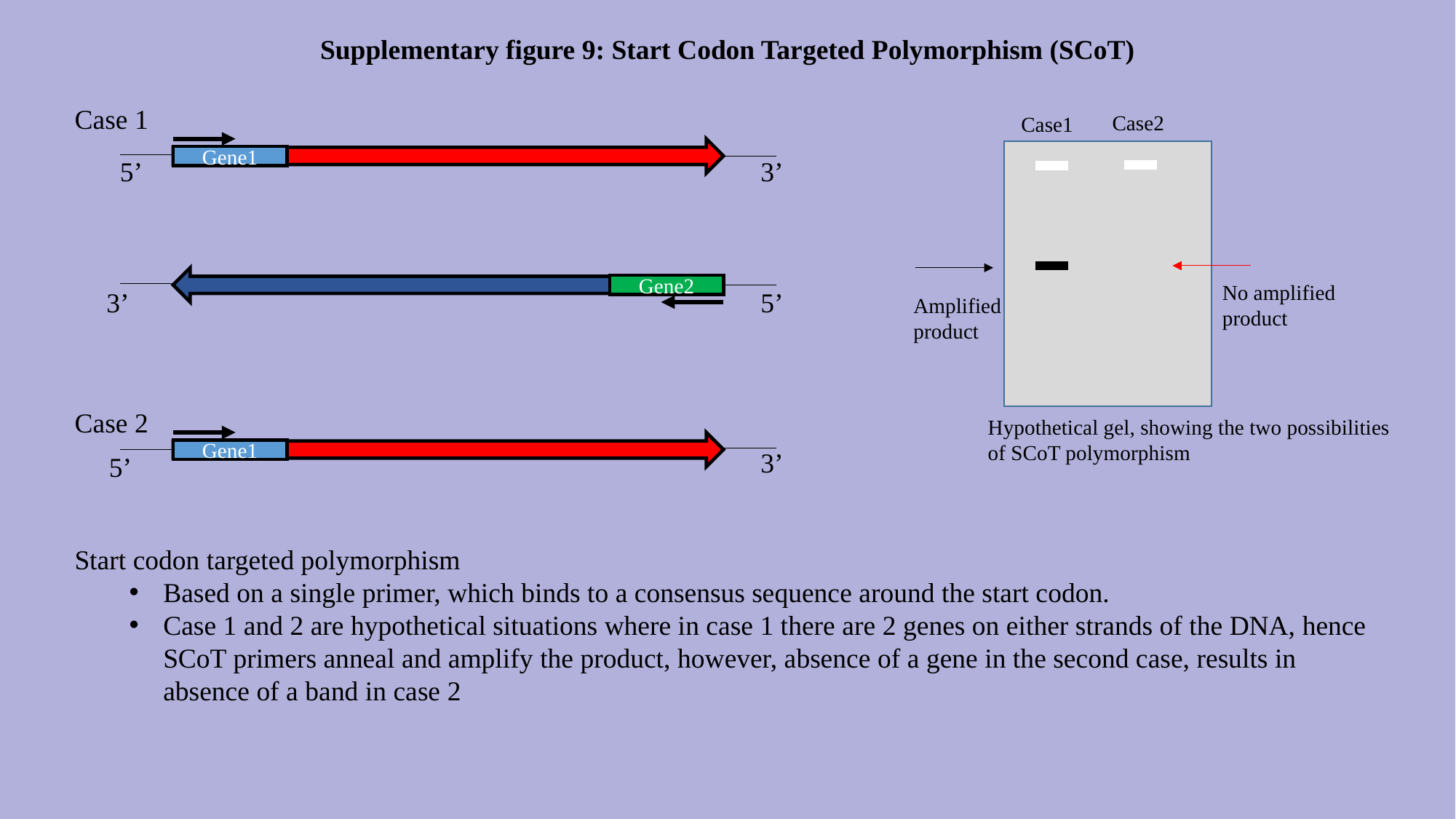

Supplementary figure 9: Start Codon Targeted Polymorphism (SCoT)
Case 1
Case2
Case1
Gene1
5’
3’
No amplified product
Gene2
3’
5’
Amplified product
Case 2
Hypothetical gel, showing the two possibilities of SCoT polymorphism
Gene1
3’
5’
Start codon targeted polymorphism
Based on a single primer, which binds to a consensus sequence around the start codon.
Case 1 and 2 are hypothetical situations where in case 1 there are 2 genes on either strands of the DNA, hence SCoT primers anneal and amplify the product, however, absence of a gene in the second case, results in absence of a band in case 2

## Slide 11
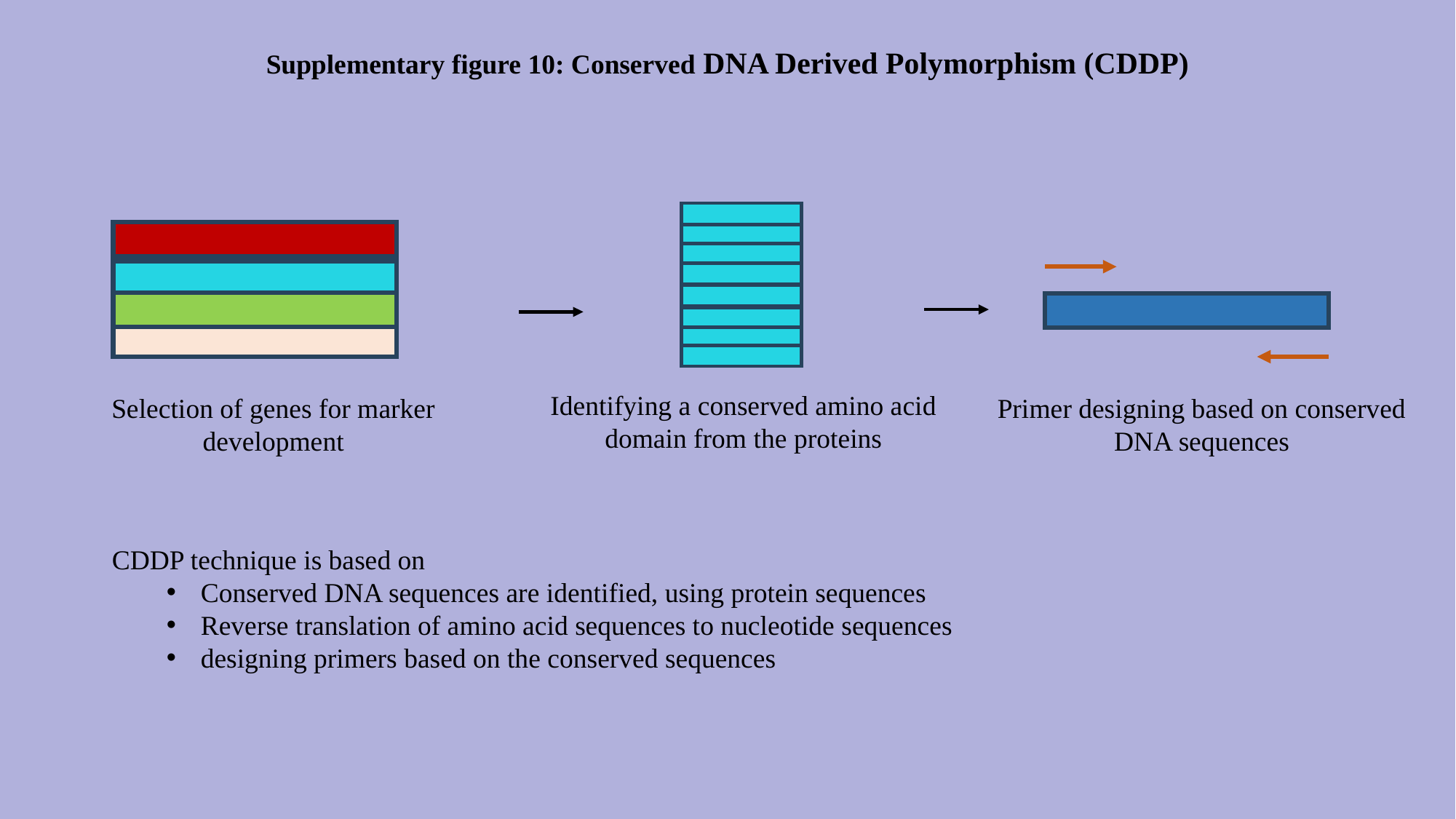

Supplementary figure 10: Conserved DNA Derived Polymorphism (CDDP)
Primer designing based on conserved DNA sequences
Identifying a conserved amino acid domain from the proteins
Selection of genes for marker development
CDDP technique is based on
Conserved DNA sequences are identified, using protein sequences
Reverse translation of amino acid sequences to nucleotide sequences
designing primers based on the conserved sequences

## Slide 12
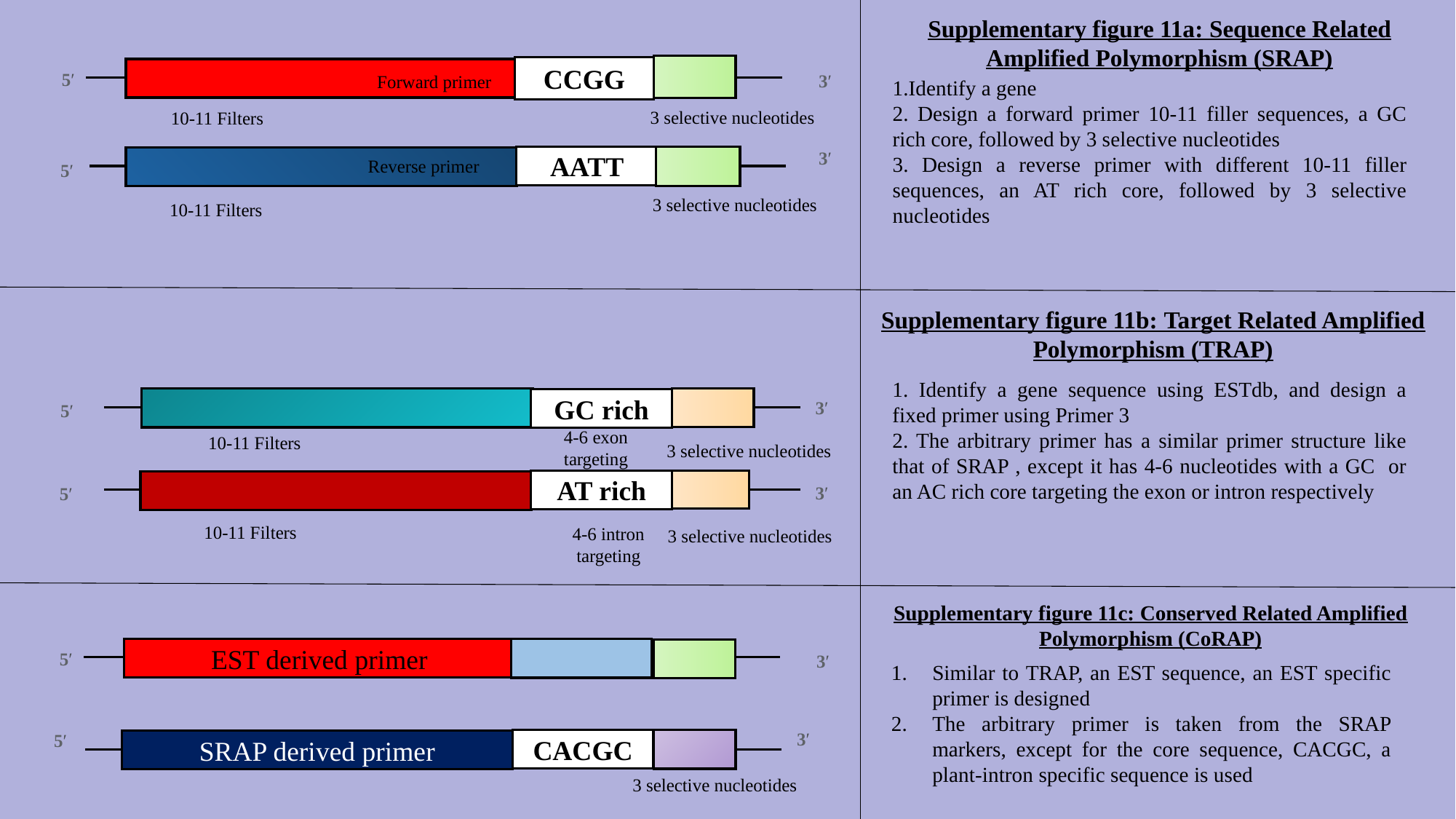

Supplementary figure 11a: Sequence Related Amplified Polymorphism (SRAP)
CCGG
5′
3′
Forward primer
1.Identify a gene
2. Design a forward primer 10-11 filler sequences, a GC rich core, followed by 3 selective nucleotides
3. Design a reverse primer with different 10-11 filler sequences, an AT rich core, followed by 3 selective nucleotides
3 selective nucleotides
10-11 Filters
3′
AATT
Reverse primer
5′
3 selective nucleotides
10-11 Filters
Supplementary figure 11b: Target Related Amplified Polymorphism (TRAP)
1. Identify a gene sequence using ESTdb, and design a fixed primer using Primer 3
2. The arbitrary primer has a similar primer structure like that of SRAP , except it has 4-6 nucleotides with a GC or an AC rich core targeting the exon or intron respectively
GC rich
3′
5′
4-6 exon targeting
10-11 Filters
3 selective nucleotides
AT rich
3′
5′
10-11 Filters
4-6 intron targeting
3 selective nucleotides
Supplementary figure 11c: Conserved Related Amplified Polymorphism (CoRAP)
EST derived primer
5′
3′
Similar to TRAP, an EST sequence, an EST specific primer is designed
The arbitrary primer is taken from the SRAP markers, except for the core sequence, CACGC, a plant-intron specific sequence is used
3′
5′
CACGC
SRAP derived primer
3 selective nucleotides

## Slide 13
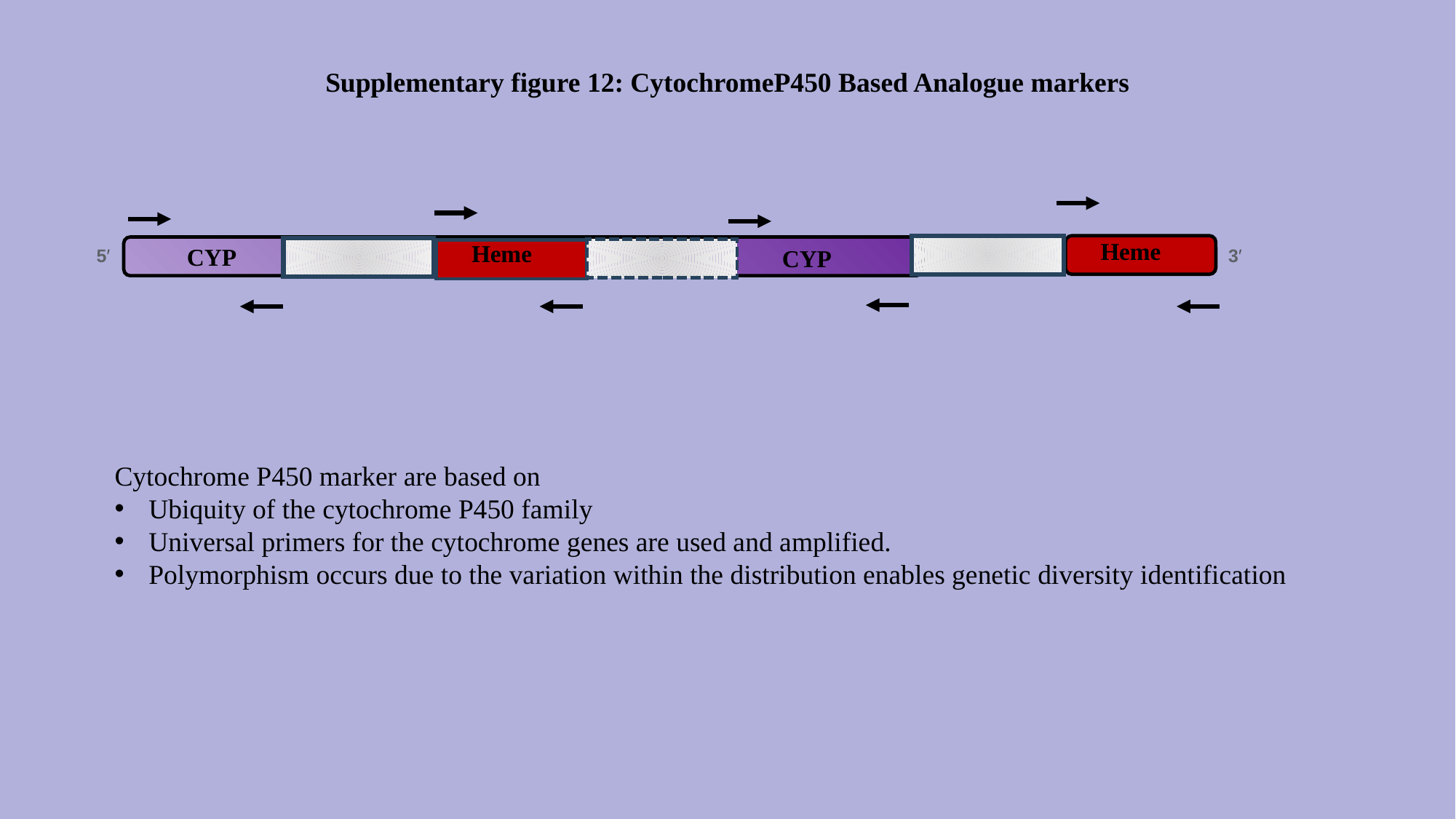

Supplementary figure 12: CytochromeP450 Based Analogue markers
Heme
Heme
CYP
CYP
5′
3′
Cytochrome P450 marker are based on
Ubiquity of the cytochrome P450 family
Universal primers for the cytochrome genes are used and amplified.
Polymorphism occurs due to the variation within the distribution enables genetic diversity identification

## Slide 14
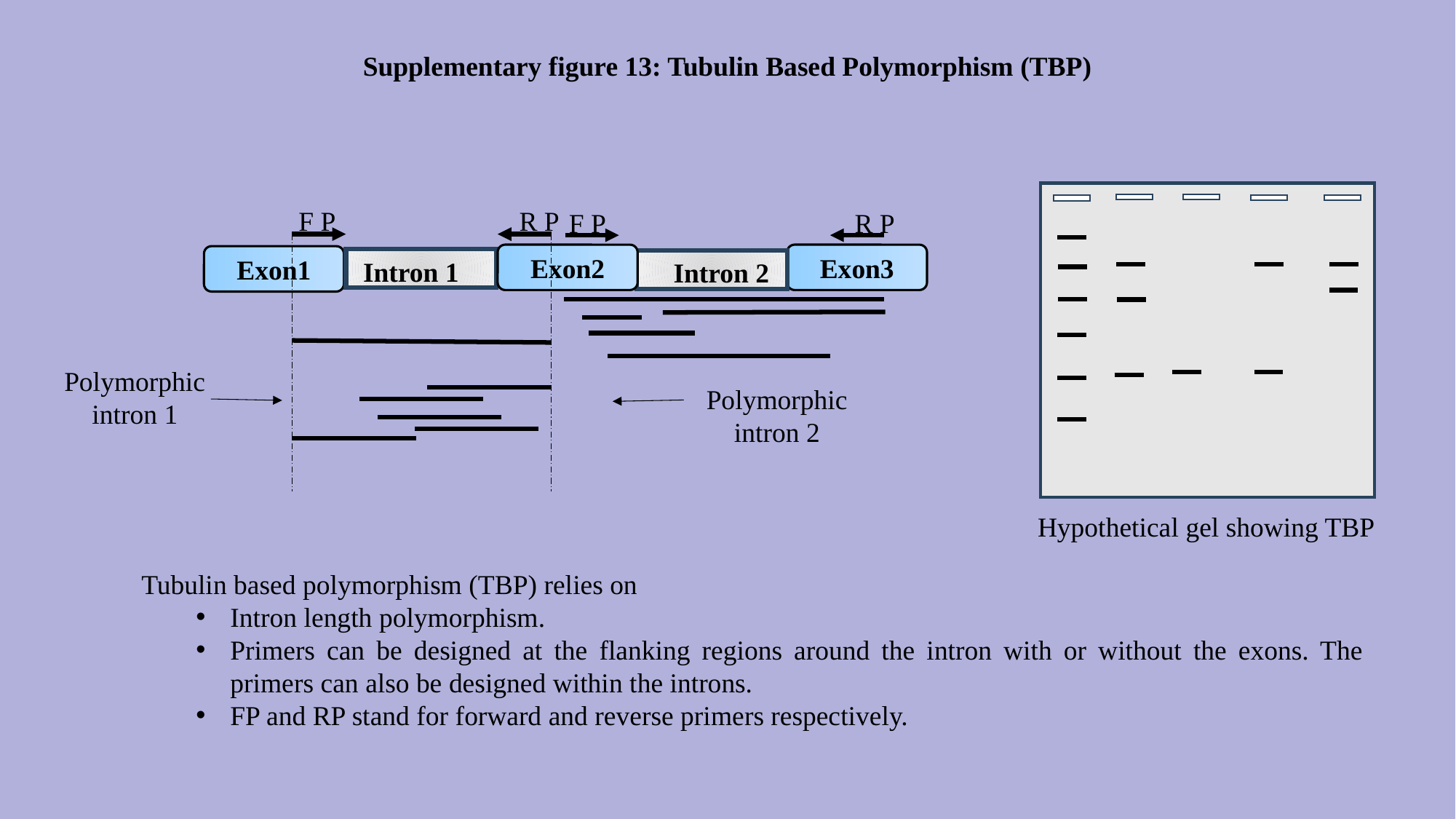

Supplementary figure 13: Tubulin Based Polymorphism (TBP)
F P
R P
F P
R P
Exon2
Exon3
Exon1
Intron 1
Intron 2
Polymorphic intron 1
Polymorphic intron 2
Tubulin based polymorphism (TBP) relies on
Intron length polymorphism.
Primers can be designed at the flanking regions around the intron with or without the exons. The primers can also be designed within the introns.
FP and RP stand for forward and reverse primers respectively.
Hypothetical gel showing TBP

## Slide 15
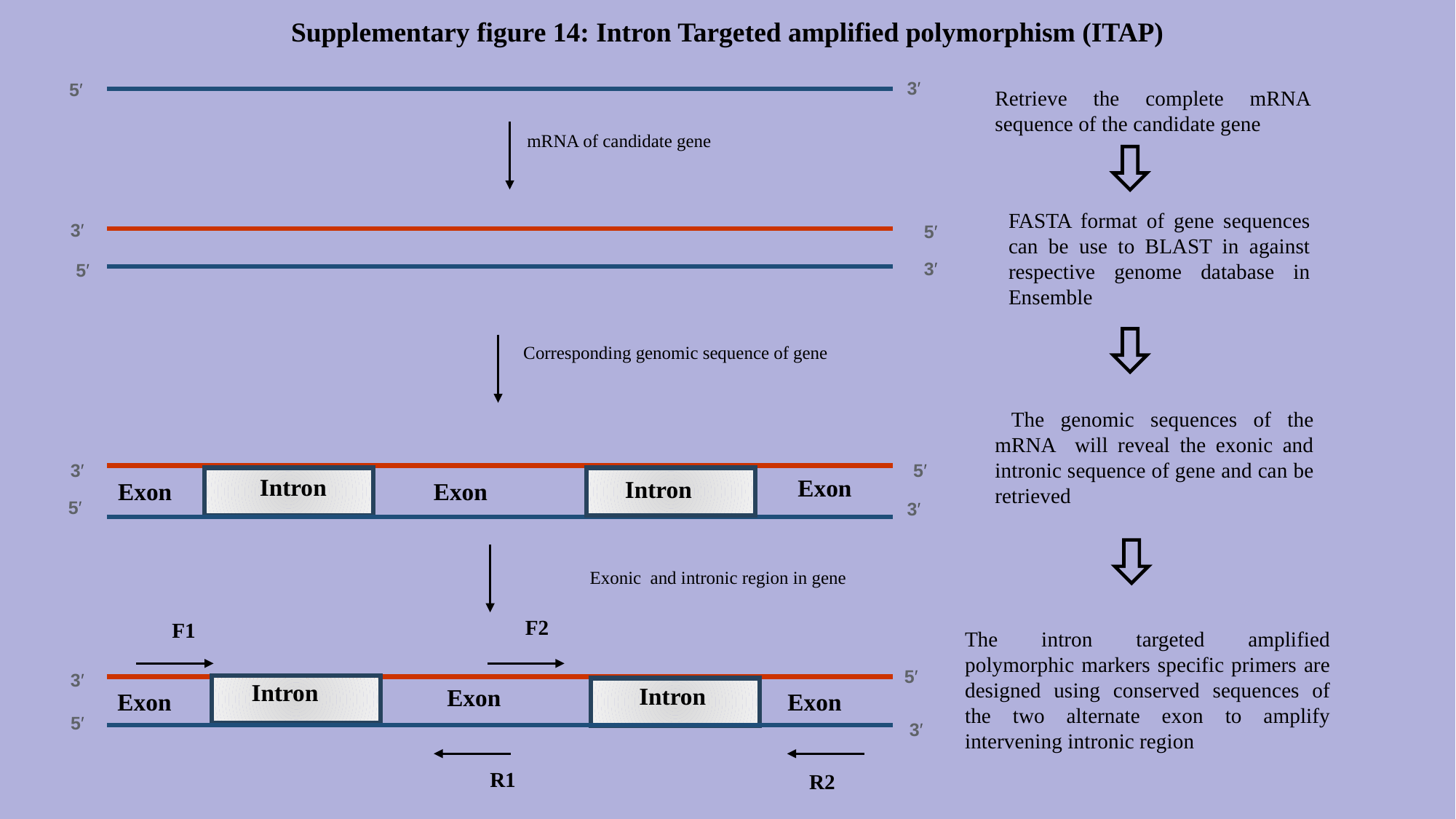

Supplementary figure 14: Intron Targeted amplified polymorphism (ITAP)
3′
5′
Retrieve the complete mRNA sequence of the candidate gene
mRNA of candidate gene
FASTA format of gene sequences can be use to BLAST in against respective genome database in Ensemble
3′
5′
3′
5′
Corresponding genomic sequence of gene
 The genomic sequences of the mRNA will reveal the exonic and intronic sequence of gene and can be retrieved
3′
5′
Intron
Exon
Intron
Exon
Exon
5′
3′
Exonic and intronic region in gene
F2
F1
The intron targeted amplified polymorphic markers specific primers are designed using conserved sequences of the two alternate exon to amplify intervening intronic region
5′
3′
Intron
Intron
Exon
Exon
Exon
5′
3′
R1
R2

## Slide 16
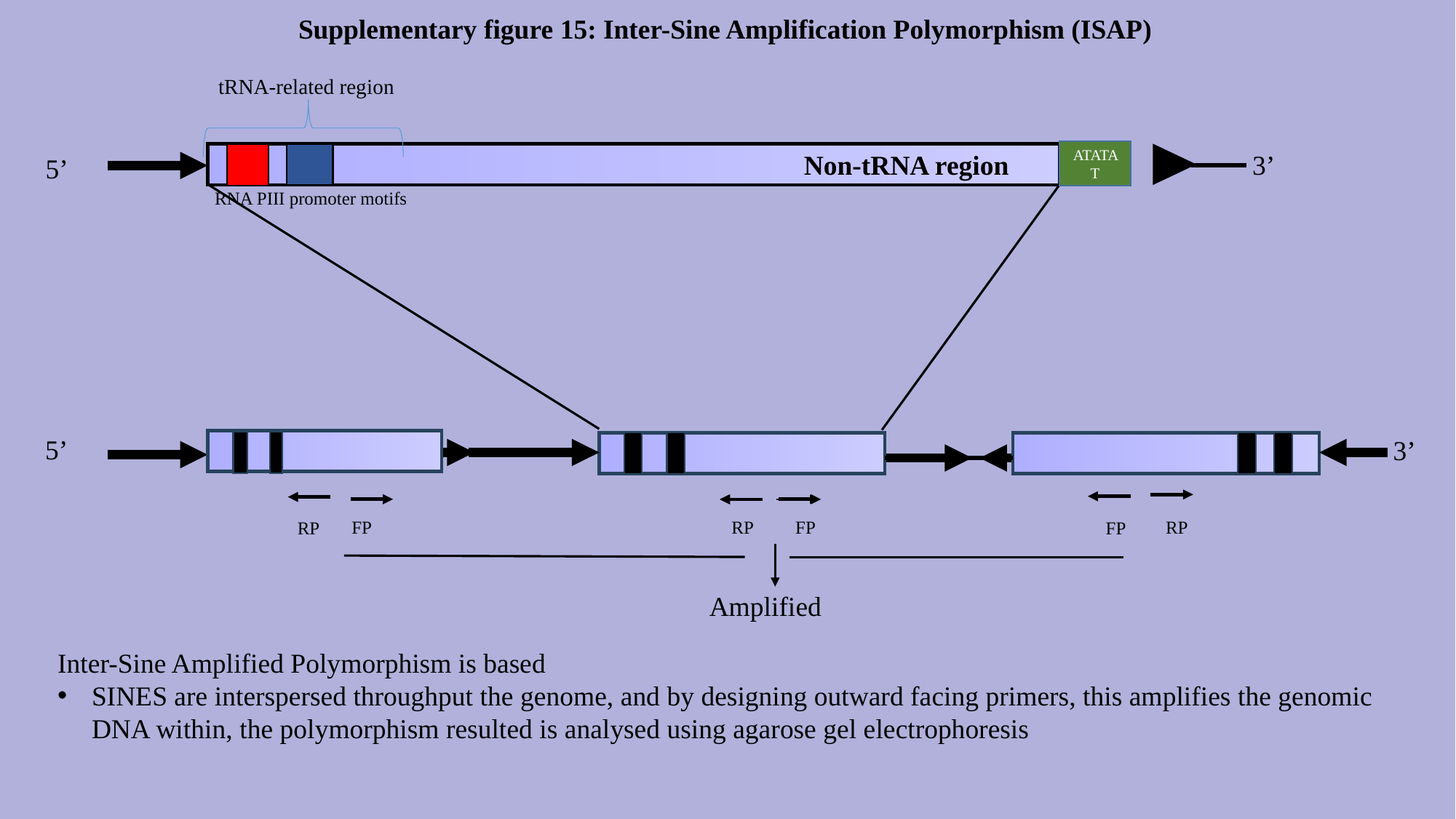

Supplementary figure 15: Inter-Sine Amplification Polymorphism (ISAP)
tRNA-related region
ATATAT
3’
 Non-tRNA region
5’
RNA PIII promoter motifs
5’
3’
RP
RP
FP
FP
RP
FP
Amplified
Inter-Sine Amplified Polymorphism is based
SINES are interspersed throughput the genome, and by designing outward facing primers, this amplifies the genomic DNA within, the polymorphism resulted is analysed using agarose gel electrophoresis

## Slide 17
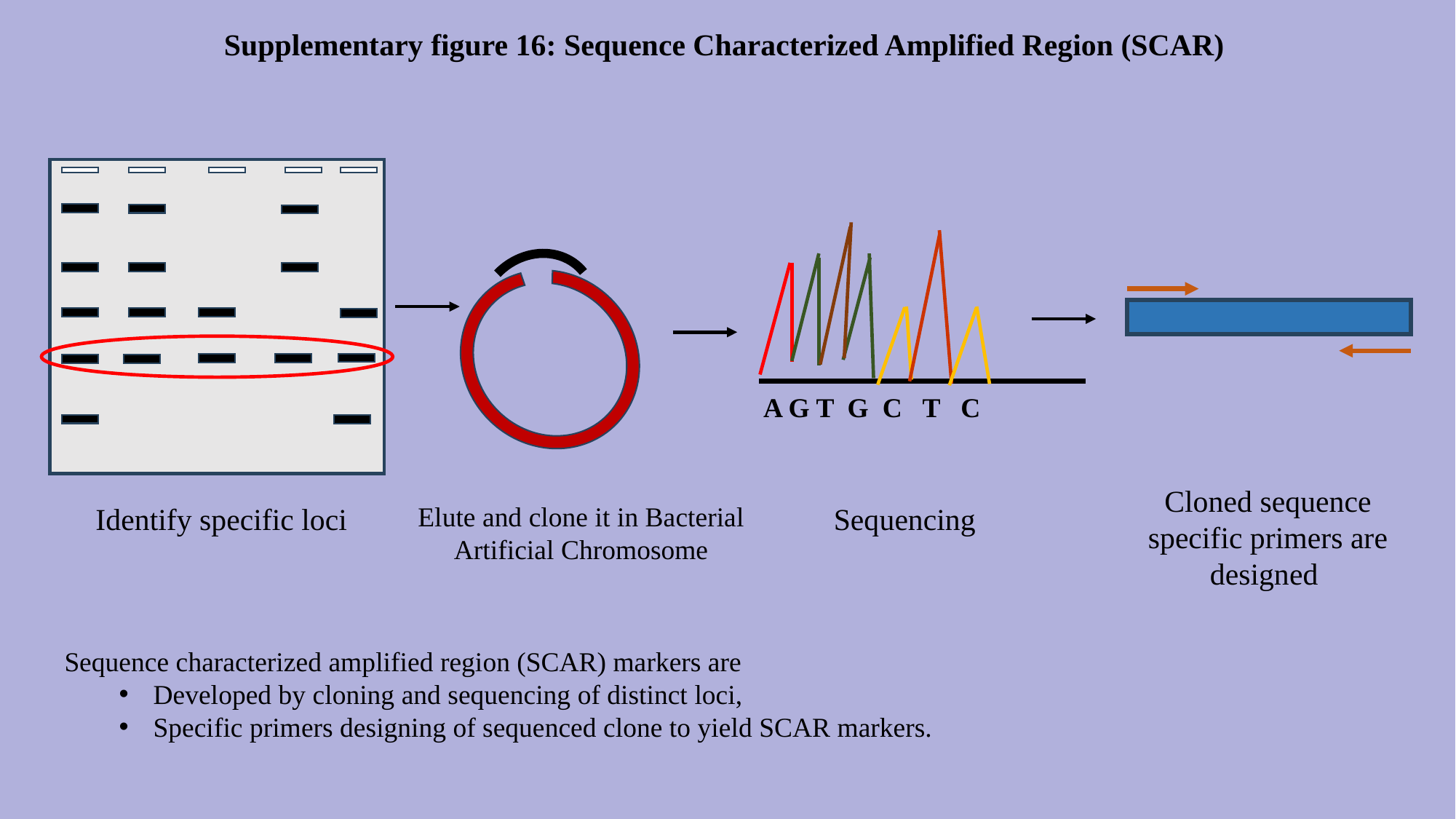

Supplementary figure 16: Sequence Characterized Amplified Region (SCAR)
A G T G C T C
Cloned sequence specific primers are designed
Elute and clone it in Bacterial Artificial Chromosome
Sequencing
Identify specific loci
Sequence characterized amplified region (SCAR) markers are
Developed by cloning and sequencing of distinct loci,
Specific primers designing of sequenced clone to yield SCAR markers.

## Slide 18
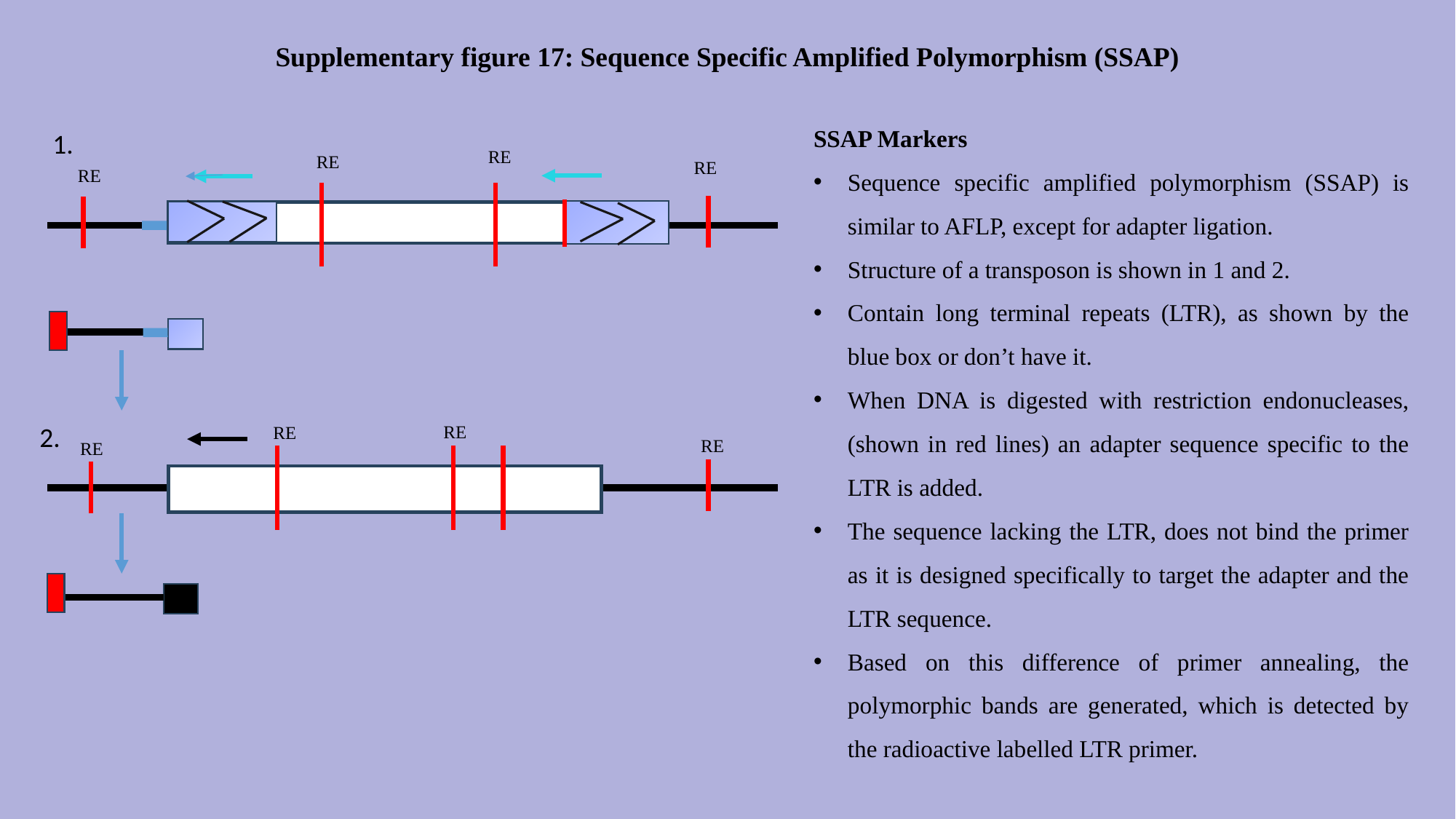

Supplementary figure 17: Sequence Specific Amplified Polymorphism (SSAP)
RE
RE
RE
RE
RE
RE
RE
RE
SSAP Markers
Sequence specific amplified polymorphism (SSAP) is similar to AFLP, except for adapter ligation.
Structure of a transposon is shown in 1 and 2.
Contain long terminal repeats (LTR), as shown by the blue box or don’t have it.
When DNA is digested with restriction endonucleases, (shown in red lines) an adapter sequence specific to the LTR is added.
The sequence lacking the LTR, does not bind the primer as it is designed specifically to target the adapter and the LTR sequence.
Based on this difference of primer annealing, the polymorphic bands are generated, which is detected by the radioactive labelled LTR primer.
1.
2.

## Slide 19
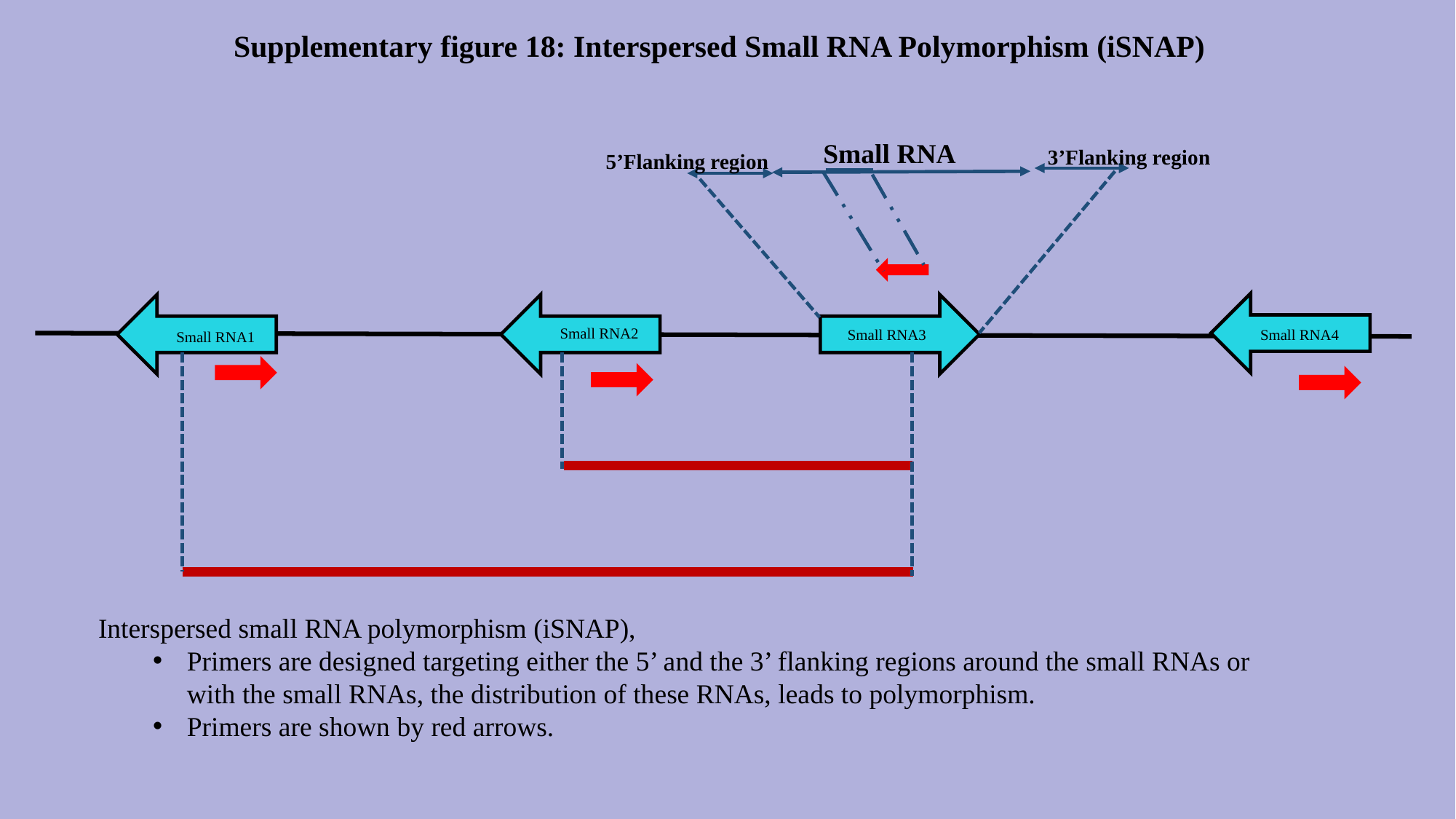

Supplementary figure 18: Interspersed Small RNA Polymorphism (iSNAP)
5’Flanking region
Small RNA
3’Flanking region
Small RNA2
Small RNA3
Small RNA4
Small RNA1
Interspersed small RNA polymorphism (iSNAP),
Primers are designed targeting either the 5’ and the 3’ flanking regions around the small RNAs or with the small RNAs, the distribution of these RNAs, leads to polymorphism.
Primers are shown by red arrows.

## Slide 20
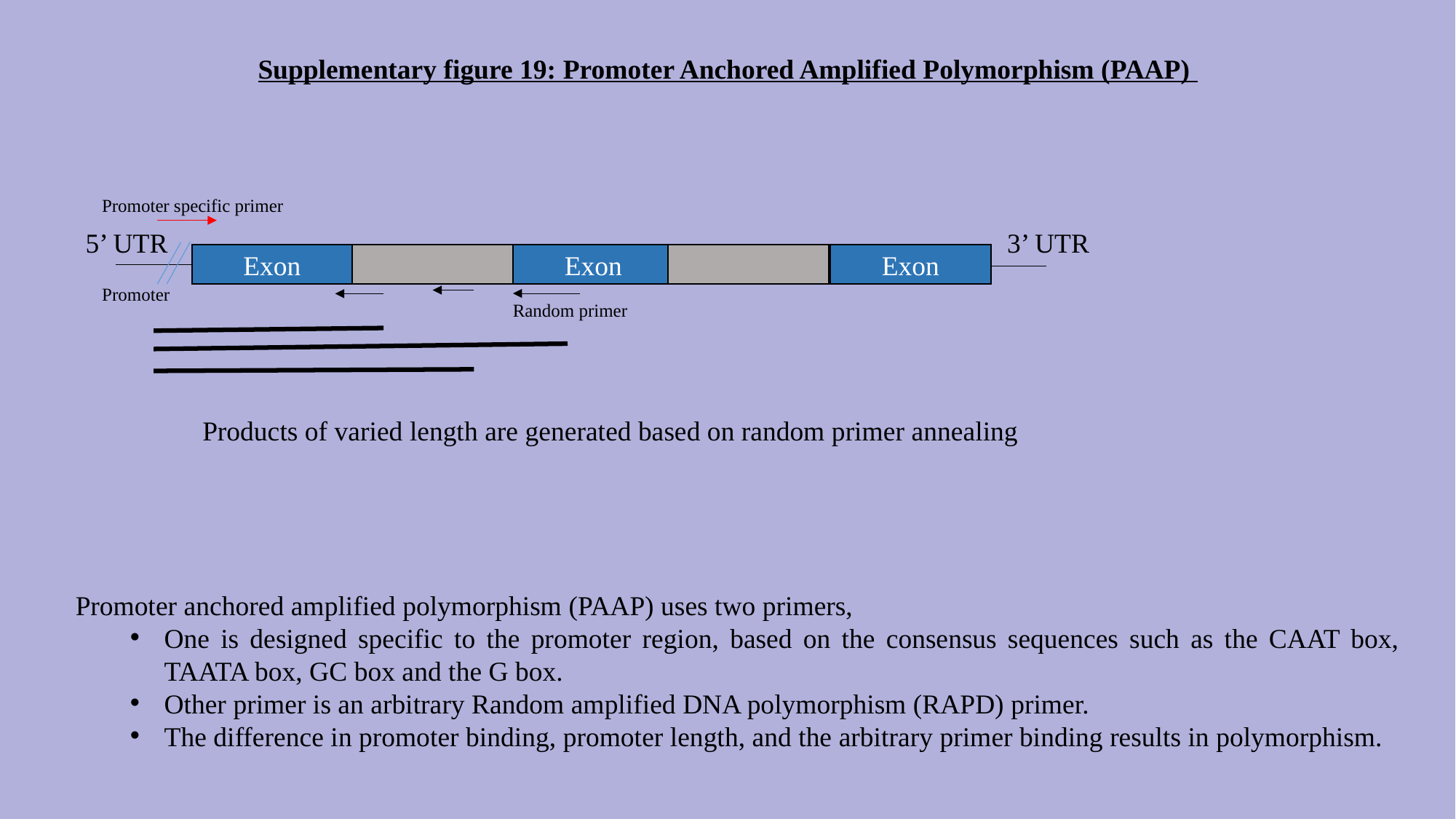

Supplementary figure 19: Promoter Anchored Amplified Polymorphism (PAAP)
Promoter specific primer
5’ UTR
3’ UTR
Exon
Exon
Exon
Promoter
Random primer
Products of varied length are generated based on random primer annealing
Promoter anchored amplified polymorphism (PAAP) uses two primers,
One is designed specific to the promoter region, based on the consensus sequences such as the CAAT box, TAATA box, GC box and the G box.
Other primer is an arbitrary Random amplified DNA polymorphism (RAPD) primer.
The difference in promoter binding, promoter length, and the arbitrary primer binding results in polymorphism.
